# Multisensory integration of affective faces and voices in psychosis proneness

**DOI:** 10.1038/s41537-025-00676-0

**Published:** 2025-09-22

**Authors:** Andreas Weiss, Patrick Bruns, Brigitte Röder, Tania M. Lincoln

**Affiliations:** 1 Institute of Psychology, Biological Psychology and Neuropsychology, University of Hamburg, Hamburg, Germany; 2 Institute of Psychology, Clinical Psychology and Psychotherapy, University of Hamburg, Hamburg, Germany; 3 LV Prasad Eye Institute, Hyderabad, Telangana India

**Keywords:** Psychosis, Schizophrenia

## Abstract

It has been proposed that dysfunctions in emotional multisensory integration (MSI) could contribute to the development of psychosis. To further substantiate this proposition, we investigated whether impaired MSI of emotional cues can be observed in people with high psychosis proneness without a diagnosis of psychosis and whether it is associated with aberrant perception and psychotic experiences. Adults scoring high vs. low on the positive subscale of the Community Assessment of Psychic Experiences (score ≥9 or <9, respectively; *n* = 36 each) categorized the perceived emotion and rated the intensity of unimodal, bimodal emotionally congruent and bimodal emotionally incongruent dynamic face-voice stimuli. In different blocks, participants were asked to attend to one modality and to ignore the other modality input. Additionally, participants completed self-report questionnaires on anomalous perceptual experiences, hallucinations and paranoia. Participants with high and low psychosis proneness did not differ in emotion categorization performance as indicated by similar inverse efficiency (IE) scores (i.e., mean reaction time divided by accuracy) in all conditions, nor did they differ in intensity ratings in any condition. Correlation analyses did not reveal significant associations between crossmodal (in)congruency effects and self-reported anomalous perceptual experiences, hallucinations or paranoia. Our findings, thus, do not provide support for the assumption that MSI of emotional cues is linked to altered perception or subclinical psychotic symptoms, nor for the notion that MSI of emotional cues is already altered at a very early stage in the developmental trajectory of psychosis.

## Introduction

Adequate processing of emotional information in our environment is essential for social functioning. For example, it is crucial to correctly identify the emotion expressed by one’s conversation partner. Already small misinterpretations of emotional cues can result in interpersonal difficulties. It is well-documented that psychosis is associated with impaired unisensory emotion processing. Studies have repeatedly demonstrated problems in recognizing the emotional meaning of face expressions and vocal prosody in patients with the diagnosis of a psychotic disorder (for reviews see^1–4^). Aberrant processing of emotional content of face or voice stimuli has been shown to be associated with psychotic symptoms such as hallucinations and delusions^5^, as well as with problems in social functioning^6,7^. These impairments in emotion recognition have additionally been observed in people with high psychosis proneness^8,9^ and in first-degree relatives^10^. In fact, impaired abilities to recognize emotions have been found to predict the transition from an at-risk status to a clinical manifestation of a psychotic disorder^11^, suggesting a potential role in the development of psychosis.

Whereas most of the previous research on emotion recognition in psychosis focused on individual sensory systems such as vision or audition, emotional information in everyday life is usually conveyed via multiple sensory systems. It has previously been shown that psychosis might be associated with a higher impairment in processing crossmodal than unimodal information (for reviews see^12–14^). This highlights the importance of investigating multisensory integration (MSI) of emotional cues in psychosis and its potential role in the development of the disorder. For example, Giannitelli et al.^15^ investigated unisensory and multisensory emotion processing in early onset psychosis. They presented affective unimodal face and voice stimuli as well as emotionally congruent crossmodal face-voice stimuli. Patients with early-onset psychosis and healthy controls had to match the perceived emotional category out of six possibilities. Patients compared to healthy controls showed significant performance impairments in all three conditions, especially for negative emotions. This suggests that uni- and multisensory emotion processing impairments might exist at the onset of psychosis. Similar results were obtained in a study by Mangelinckx et al.^16^, in which patients with the diagnosis of a psychotic disorder and healthy controls were required to identify the emotions of face and voice stimuli in five different conditions: unimodal visual, unimodal auditory, emotionally congruent audiovisual, emotionally incongruent audiovisual attend the face and emotionally incongruent audiovisual attend the voice. Mangelinckx et al.^16^ observed an impaired emotion categorization performance in patients compared to controls over all five conditions. Although these findings suggest a general emotion processing deficit in psychosis, the overall empirical findings on MSI of emotional cues in psychosis are inconsistent^12,17^ with some pointing to impaired^16,18–25^, some to enhanced^19,26^, and some to typical^27–30^ MSI of emotional cues. Crossmodal congruency effects typically consist of benefits for emotional categorization if a face expression and the accompanying affective prosody match and impairments in performance in case they do not match^31,32^.

Other limitations of currently available case-control studies are small and heterogeneous samples with regard to symptom profiles, severity, and medication status. Thus, it is unknown whether impaired MSI of emotional cues is a consequence or cause of psychotic disorder. Here, we investigated individuals exhibiting an increased vulnerability for psychosis but without a clinically manifested psychotic disorder. Impaired MSI of emotional cues in this group would hint towards multisensory processing deficits contributing to, rather than following, cognitive processing deficits in patients with psychotic disorders.

Another limitation of the cross-sectional research on MSI of emotional cues in psychosis is the sparse mechanistic understanding of how altered MSI of emotional cues might contribute to psychosis. Opoku-Baah et al.^33^ hypothesized that a failure to appropriately integrate crossmodal social cues could lead to a fragmented representation of environmental social information, exacerbating emotional processing deficits and thereby impacting social functioning. Postmes et al.^34^ suggested that impaired MSI of external and proprioceptive cues might facilitate aberrant percepts since the sensory consequences of one’s own actions and other external sensory events could not be properly distinguished^35^. As a consequence, unusual, irritating and attention capturing sensations of overly increased intensity would become more likely^36,37^. Delusional interpretations might be a consequence of the attempt to account for such aberrant percepts^38^.

Here we tested for potential links between dysfunctional MSI of emotional cues, anomalous perceptual experiences and psychotic symptoms in the same participants. We predicted that aberrant MSI of emotional cues is associated with the presence of anomalous perceptual experiences in subclinical, psychosis-prone individuals. We anticipated that associations between MSI of emotional cues and psychotic symptoms are stronger for perceptual symptoms such as hallucinations than for more distal, cognitive symptoms such as paranoia.

Participants with low vs. high psychosis proneness had to categorize the emotion of facial expressions and vocal prosody. Stimuli were presented unimodally or bimodally. In the latter condition, the emotion of the facial expression either matched (congruent condition) or did not match (incongruent condition) the vocal prosody^32^. In the bimodal condition, participants were asked to attend to one sensory modality and to ignore the input of the other modality. Thus, an implicit and automatic MSI was assessed. We expected participants with a high psychosis proneness to show altered (in)congruency effects during audiovisual emotion processing compared to participants with low psychosis proneness, which would be indicative of atypical MSI of emotional cues in subclinical psychosis. Further, we expected significant correlations between (in)congruency effects and self-reported anomalous perceptual experiences which would suggest a direct (but yet unexplored) link between perceptual anomalies and experimentally assessed atypical MSI of emotional cues.

## Methods

### Participants

We recruited participants from the local community of the city of Hamburg and amongst students of the University of Hamburg. After an initial contact, participants received a link to an online screening, which allocated them to either the high or the low psychosis proneness group (see 2.2 Procedure). Participants with an acute or past diagnosis of a mental disorder, which we assessed via self-report (see Supplement 1), including psychotic disorders, bipolar disorder or autism, as well as acute suicidality, neurological disorders, regular and/or recent consumption of psychedelic substances, uncorrected sight or hearing impairment or inability to follow task instructions were excluded. Since previous evidence indicated a higher prevalence of substance abuse^39^, anxiety^40^ and depression^41^ in samples with psychosis proneness, we did not exclude subjects with mild forms of either of these disorders.

Individuals who fulfilled these criteria were invited to the experimental session. The final sample consisted of *N* = 72 participants, with *n* = 36 in the high and *n* = 36 in the low proneness group (see Table 1 for demographic data). Participants gave written informed consent prior to participation and received 10€/hour or course credits as compensation. This study was approved by the Local Ethics Committee of the University of Hamburg.

**Table 1:** Demographic and diagnostic data and test statistics of group comparisons.

	High proneness	Low proneness	
*n*	36	36	
Age, *M* (range)	24.8 (19–39)	25.9 (18–42)	*t*(70) = –0.87, *p* = 0.385, Cohen’s *d* = 0.21
Gender, *n* (%)			
Female	29 (80.6)	26 (72.2)	*Χ*^*2*^ = 2.16, *p* = 0.339, *φ* = 0.17
Male	6 (16.7)	10 (27.8)	
Diverse	1 (2.8)	0	
Education, *n*			
10 years	1	2	*Χ*^*2*^ = 3.01, *p* = 0.222, *φ* = 0.21
12–13 years^a^	27	20	
University degree^b^	8	14	
CAPE positive, *M* (*SD*)	14.0 (6.2)	4.3 (2.1)	
CAPS, *M* (*SD*)	11.3 (6.4)	5.7 (4.3)	*t*(61.52) = –4.31, ***p*** < **0.001**, Cohen’s *d* = 1.02
LSHS-E, *M* (*SD*)	15.3 (9.7)	8.6 (7.1)	*t*(70) = –3.39, ***p*** = **0.001**, Cohen’s *d* = 0.80
PCL, *M* (*SD*)^c^	47.3 (27.1)	34.8 (26.3)	*t*(70) = –1.89, *p* = 0.052, Cohen’s *d* = 0.47

*N* = 72. *p*-values < 0.05 are marked bold.

*CAPE positive* Community Assessment of Psychic Experiences, positive subscale, *CAPS* Cardiff Anomalous Perception Scale, total score, *LSHS-E* Launey-Slade Hallucination Scale-Extended, total score, *PCL* Paranoia Checklist, total score.

^a^Due to changes in German education law in 2007, the years of education for acquiring the Abitur (A-level/high school equivalent) vary between 12 and 13 years depending on school and Federal state.

^b^University degree: Bachelor level or higher.

^c^One participant in the low and one in the high proneness group had ≥50% missings in the Paranoia Checklist. Their Paranoia Checklist scores were corrected by the respective group mean.

A sensitivity analysis carried out in G*Power 3.1.9.6^42^ indicated that the sample size of *N* = 72 had 80% power (at a conventional α-level of 0.05) to detect a medium-sized (*η*^*2*^_*p*_ = 0.07) ANOVA interaction effect of *Group* (high vs. low proneness) and *Stimulus Condition* (unimodal vs. bimodal emotionally congruent vs. bimodal emotionally incongruent) in our main analysis. Previous studies which had reported altered MSI of emotional cues in patients with psychotic disorders typically observed medium-to-large effect sizes (*η*^*2*^_*p*_ ≥ 0.06) for the interaction effect of Group and Experimental Condition^19,20,22,23^. Thus, our study was sufficiently powered to detect effects of similar size.

### Procedure

Prior to the experimental session, participants completed an online screening which collected demographic data and screened for inclusion and exclusion criteria. In addition, we administered the 28-item version of the Community Assessment of Psychic Experiences (CAPE)^43^, a self-report questionnaire on life-time occurrence of psychotic symptoms, as a measure for psychosis proneness. Analogous to previous research^44^, we applied a cut-off value of ≥9 on the positive subscale of the CAPE to assign participants to one of two subgroups (high proneness ≥9 vs. low proneness <9 for psychotic disorder). This cut-off was selected to obtain a high proneness sample with CAPE positive scores that are outside the range of healthy samples with no clinical disorder (*M* = 3.8, SD = 3.4^45^) and above the mean of large community samples (*M* = 8.2, SD = 5.2)^46^ (adjusted for scoring procedures).

The experimental session took place in a sound attenuated and dimly lit room at the University of Hamburg. Prior to the experimental run, baseline diagnostic measures were collected. These included personal and family history of psychotic disorders, regular medication, recent substance consumption, sight or hearing impairment and respective corrections and handedness.

It has previously been shown that psychosis proneness is associated with difficulties in attention and vigilance (for reviews and meta-analyses see^47,48^). To be able to rule out that possible differences in the MSI of emotional cues between individuals with high and low psychosis proneness are attributable to difficulties adhering to the experimental procedure, we employed the Trail-Making-Test (TMT) Version A and B^49^ to assess attention and the Continuous Performance Test - Identical Pairs (CPT-IP)^50^ to assess vigilance.

Subsequently, participants completed the experimental paradigm on audiovisual emotion processing, adopted from Föcker et al.^32^, using the Software Presentation (Version 21.1; Neurobehavioural Systems Inc., Berkeley, California, USA). In total, 216 emotionally laden audiovisual and 108 unimodal stimuli were presented centrally on a 24-inch computer screen (screen resolution: 1920 × 1200) at 65 cm viewing distance (4° width and 9° height of visual angle) and via computer screen speakers (60 to 65 dB(A), measured at the participants’ head position). Stimuli consisted of visual and auditory recordings of 4 actors (2 female, 2 male) uttering disyllabic German pseudowords (“lolo”, “tete”, “gigi”). Visual stimuli consisted of video recordings of the actor’s facial features during pseudoword utterance, auditory stimuli of the auditory stream (for stimulus creation and selection, see^32^). Facial expressions and affective prosody featured one of four emotions: happy, angry, sad or neutral. Stimuli were either unimodal visual, unimodal auditory, bimodal emotionally congruent or bimodal emotionally incongruent, with 48 stimuli per stimulus condition. Additionally, visual and auditory deviant stimuli (6 per stimulus condition) were introduced to guarantee that participants paid attention to the stimuli at all times. Visual deviants included a black dot (0,6°/6 mm diameter, duration 100 ms) in one of four possible locations (forehead, nose, left or right cheek) during the last 130–330 ms of the video; auditory deviants included one of four tones (600, 900, 1200 or 1500 Hz, duration 100 ms) during the last 130–330 ms of the auditory stream.

**Fig. 1:**
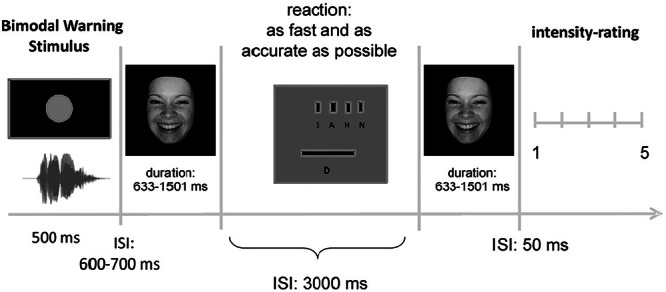
Experimental design. Capital letters represent buttons for emotion categorization: S sad, A angry, H Happy, N Neutral, D deviant recognition via space bar. Adopted from “Preattentive processing of audio-visual emotional signals” by J. Föcker, M. Gondan, & B. Röder, 2011, *Acta Psychologica, 137*(1), 36–47 (10.1016/j.actpsy.2011.02.004).

In each trial, after a bimodal warning stimulus (grey circle with 2° of visual angle combined with multispeaker noise, duration = 500 ms), each stimulus was presented twice after a random interstimulus interval (600–700 ms, uniform distribution; see Fig. 1 for a depiction of the trial structure).

Following the first onset, participants had to categorize the perceived emotion as fast and as accurately as possible by button press on a standard keyboard. Each of four buttons was assigned to one of the four emotions as well as to one finger of the right hand (index to little finger). The assignment order of emotion category to the buttons was counterbalanced across participants. After an interstimulus interval of 3000 ms, the same stimulus was presented a second time. After stimulus offset, a rating scale appeared on the screen and participants had to rate the intensity of the perceived emotion from 1 (very low) to 5 (very high) by pressing the respective key (1–5) on the keyboard. Immediately after the intensity rating, the next trial started. In case of deviant stimuli, participants had to press the space bar following the first presentation to indicate that they recognized the deviant. After pressing the space bar, the next trial started immediately.

Stimuli were divided into 12 blocks of 27 stimuli each (8 unimodal, 8 bimodal congruent, 8 bimodal incongruent and 3 deviant stimuli) which were presented in randomized order. For half of the blocks, participants had to attend to the face while ignoring the voice and vice versa. Unimodal visual and auditory stimuli occurred only in blocks in which the respective modality had to be attended. The order of blocks alternated between attend to face and attend to voice, with half of the participants starting with the attend face and the other half with the attend voice condition. After each block, participants had the opportunity to take a short break of 2–3 min. Before the start of the actual experiment, 10 practice trials were conducted to familiarize participants with the task. The experiment took ~50 min to complete.

After the experimental run, participants completed the following self-report instruments: The Cardiff Anomalous Perception Scale (CAPS)^36^ for measuring anomalous perceptual experiences, the Launey-Slade Hallucination Scale-Extended (LSHS-E)^51,52^ for measuring hallucinatory experiences and the Paranoia Checklist^53^ for measuring paranoid thoughts and convictions. The Brief Core Schema Scale^54^ was additionally administered to explore whether it moderates the effect of altered MSI on clinical manifestations, but is of no relevance for the present manuscript.

### Data analysis

To examine group differences in demographic data, we calculated an unpaired *t*-test for age and *Χ*^2^-tests for gender and education level (see Table 1 for test statistics).

For each participant, experimental data were preprocessed using RStudio Version 1.0.143 (RStudio Team, 2016) by calculating mean reaction times (RT, in ms) and accuracy (proportion of correct emotion categorizations) for categorization data as well as mean perceived emotional intensity for intensity ratings. These calculations were performed separately per stimulus condition (unimodal, bimodal congruent and bimodal incongruent) and attention condition (attend face vs. attend voice). Incorrectly categorized stimuli and trials with RTs below 200 ms or above 3950 ms were excluded from the analyses. Deviant trials were analyzed separately (see below). To account for a possible speed-accuracy trade-off, we calculated inverse efficiency (IE) scores for categorization data by dividing mean RT by accuracy^55^. Statistical analyses were performed using IBM SPSS Statistics for Windows, Version 27.0 (IBM Corp., 2020).

To examine whether participants followed task instructions, we calculated the overall percentage of recognized deviants for each participant. If less than 60% of the deviants were detected, the participant was excluded from further analyses (adopted from Föcker et al.^32^). Further, we calculated an unpaired *t*-test to check for group differences in deviant recognition.

IE scores were analyzed separately for attend face and attend voice by means of mixed ANOVAs with *Group* (high vs. low proneness) as between-subjects factor and *Stimulus Condition* (unimodal vs. bimodal emotionally congruent vs. bimodal emotionally incongruent) as within-subjects factor (for an analysis with attention as additional within-subjects factor, see Supplement 2). The Huynh-Feldt correction^56^ was applied in case of sphericity violations. Post-hoc pairwise comparisons were calculated by means of Bonferroni corrected *t*-tests. The same analyses were run for mean intensity ratings. Additionally, we report results of equivalent analyses for mean RT and accuracy (see Supplement 3) and exploratory emotion-specific analyses of RT and accuracy (see Supplement 4).

Questionnaire scores (LSHS-E, CAPS and the Paranoia Checklist) were each compared between the high and low proneness group by means of unpaired *t*-tests (see Table 1 for test statistics).

In order to investigate associations between (in)congruency effects in the experimental task and questionnaire scores, IE difference scores between each pairing of stimulus conditions were calculated: congruent–unimodal, unimodal–incongruent, and congruent–incongruent. Next, Pearson’s correlation coefficients (*r*) between IE difference scores, CAPS, LSHS-E and Paranoia Checklist were derived. Bonferroni-correction was applied for *p*-values of correlations between questionnaire scores and the IE difference score congruent–unimodal, reflecting crossmodal facilitation, and the IE difference score unimodal–incongruent, reflecting crossmodal interference. We calculated the Pearson correlation coefficient *r*, since studies have shown that *r* can be assumed to be robust against violations of the normality distribution, especially when *n* > 30^57–59^.

A similar procedure as for IE was used for deriving difference scores in perceived emotional intensity. Subsequently, Pearson’s correlation coefficients (*r*) between intensity difference scores, CAPS, LSHS-E and Paranoia Checklist were calculated, with Bonferroni-correction as described above. Additionally, Bonferroni-corrected Pearson’s correlation coefficients (*r*) were calculated between absolute IE scores, CAPS, LSHS-E and Paranoia Checklist (see Supplement 5).

Potential group differences in attention and processing speed were analyzed by means of two unpaired *t*-tests entering TMT-A and TMT-B completion times (in seconds) as dependent variables, respectively. To check for group differences in vigilance, CPT-IP *d’* scores, i.e., sensitivity scores reflecting attentional capacity^60^, were analyzed with a mixed ANOVA with *Group* (low proneness vs. high proneness) as between-subjects factor and *Digit Load* (2 vs. 3 vs. 4 digits) as within-subjects factor, followed by post-hoc *t*-tests. In case of significant group differences in the TMT or CPT-IP, we assessed associations of TMT and CPT-IP scores with the main dependent variable of the experimental task, i.e., IE scores, by means of Pearson’s correlation coefficients (*r*).

## Results

### Demographic data

From the originally 87 participants enrolled in the study, *n* = 3 in the high and *n* = 3 in the low proneness group were excluded due to technical difficulties during data collection, *n* = 1 in the high and *n* = 5 in the low proneness group due to inability to detect ≥60% of deviants in the experimental task, *n* = 1 in the high and *n* = 1 in the low proneness group since task performance deviated more than two *SD* from the group mean, and *n* = 1 in the high proneness group due to emotion categorizing accuracy below chance level (i.e., <25%). Therefore, the datasets of *N* = 72 participants, 36 in each group, entered the analyses. Demographic data and statistical comparisons are presented in Table 1. There were no group differences in age, gender and education. Only two participants reported current anxiety (one in each group), two indicated anxiety in the last six months (one in each group), and one indicated depression in the last six months (high proneness group). No participant reported current or earlier substance abuse (see Supplement 1 for further detail). The high psychosis proneness group showed significantly higher scores in the CAPS and the LSHS and a trend towards higher scores on the Paranoia Checklist.

### Experimental data

#### Deviant recognition

Groups did not differ in percentage of recognized deviants (high proneness: *M* = 88.7%, *SD* = 10.2; low proneness: *M* = 84.6%, *SD* = 10.3; 95% CI for the difference in means: [–0.8, 8.9]), *t*(70) = –1.67, *p* = 0.099, Cohen’s *d* = 0.39.

#### Inverse efficiency analysis

##### Attend face

**Fig. 2:**
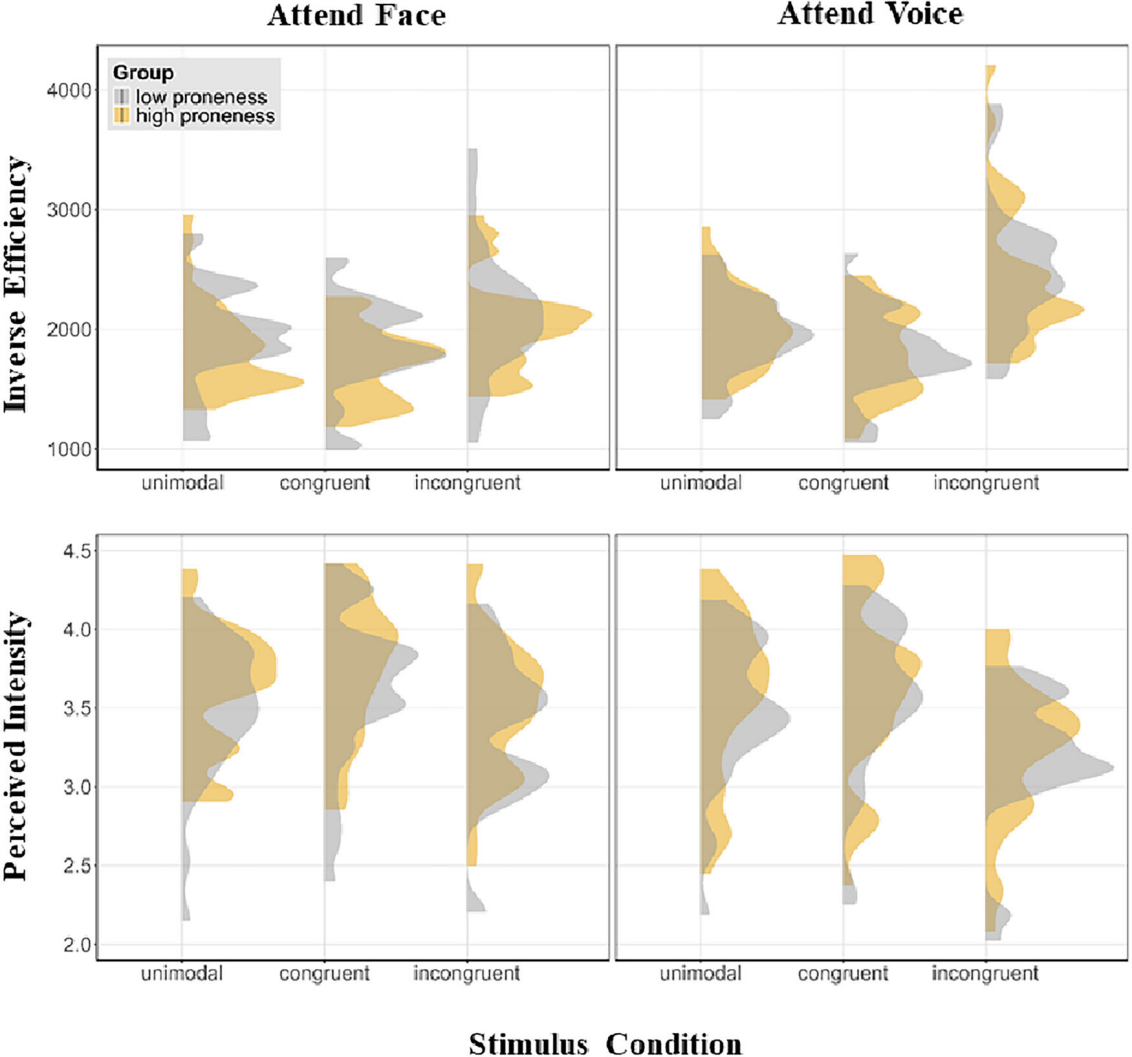
Inverse efficiency scores and perceived emotional intensity per group and stimulus condition, separated by attention condition. For both, the low and the high proneness groups (*n* = 36 each), the distributions of inverse efficiency scores and emotional intensity ratings are depicted per stimulus condition and attention condition. Congruent = bimodal emotionally congruent; incongruent = bimodal emotionally incongruent.

A significant main effect of *Stimulus Condition* (*F*(1.6, 112.1) = 35.94, *p* < 0.001, *η*^*2*^_*p*_ = 0.34) revealed lower IE scores in the congruent (*M* = 1781 ms, *SD* = 349) compared to both the unimodal (*M* = 1898 ms, *SD* = 396; 95% CI for the difference in means: [–167, –68]), *t*(71) = –4.70, *p* < 0.001, Cohen’s *d* = 0.55, and the incongruent condition (*M* = 2082 ms, *SD* = 454; 95% CI for the difference in means: [–386, –217]), *t*(71) = –7.13, *p* < 0.001, Cohen’s *d* = 0.84, as well as lower IE scores in the unimodal compared to the incongruent condition (95% CI for the difference in means: [–259, –109]), *t*(71) = –4.92, *p* < 0.001, Cohen’s *d* = 0.58. Groups did not differ in overall emotion categorization (no main effect of *Group, F*(1, 70) = 1.88, *p* = 0.175, *η*^*2*^_*p*_ = 0.03), or in (in)congruency effects during emotion categorization (no *Group* * *Stimulus Condition* interaction), *F*(1.6, 112.1) = 0.23, *p* = 0.748, *η*^*2*^_*p*_ = 0.003 (see Fig. 2 top row left for IE scores per group and stimulus condition).

In an exploratory analysis, we tested whether higher CAPE cut-off values would have changed this result pattern by evaluating nine different cut-offs (≥10 and ≤20). However, neither the main effect of *Group* (uncorrected *p* ≥ 0.079, *η*^*2*^_*p*_ ≤ 0.04) nor the *Group* * *Stimulus Condition* interaction (uncorrected *p* ≥ 0.524, *η*^*2*^_*p*_ ≤ 0.008) were significant at any of these cut-off values.

##### Attend voice

A significant main effect of *Stimulus Condition* (*F*(1.3, 88.1) = 120.59, *p* < 0.001, *η*^*2*^_*p*_ = 0.63) indicated lower IE scores in the congruent (*M* = 1800 ms, *SD* = 358) compared to both the unimodal (*M* = 1938 ms, *SD* = 327; 95% CI for the difference in means: [–226, –141]), *t*(71) = –8.64, *p* < 0.001, Cohen’s *d* = 1.02, and the incongruent condition (*M* = 2467 ms, *SD* = 544; 95% CI for the difference in means: [–776, –558]), *t*(71) = –12.19, *p* < 0.001, Cohen’s *d* = 1.44, as well as lower IE scores in the unimodal compared to the incongruent condition (95% CI for the difference in means: [–581, –385]), *t*(71) = –9.84, *p* < 0.001, Cohen’s *d* = 1.16. Groups did not differ in overall emotion categorization (no main effect of *Group*, *F*(1, 70) = 0.002, *p* = 0.964, *η*^*2*^_*p*_ < 0.001), or in (in)congruency effects during emotion categorization (no *Group* * *Stimulus Condition* interaction), *F*(1.3, 88.1) = 0.43, *p* = 0.559, *η*^*2*^_*p*_ = 0.006 (see Fig. 2 top row right for IE scores per group and stimulus condition).

The main effect of *Group* was not significant at any of the explored CAPE cut-off values of ≥10 and ≤20 (uncorrected *p* ≥ 0.085, *η*^*2*^_*p*_ ≤ 0.04). However, the *Group* * *Stimulus Condition* interaction was significant (uncorrected *p* ≤ 0.047, *η*^*2*^_*p*_ ≥ 0.05) for cut-off values of ≥18 due to higher IE scores in the high (*n* = 8) vs. low (*n* = 64) group particularly in the incongruent condition.

#### Intensity analysis

##### Attend face

A significant main effect of *Stimulus Condition* (*F*(1.9, 130.4) = 28.42, *p* < 0.001, *η*^*2*^_*p*_ = 0.29) revealed higher intensity ratings in the congruent (*M* = 3.68, *SD* = 0.46) compared to both the unimodal (*M* = 3.56, *SD* = 0.41; 95% CI for the difference in means: [0.07, 0.17]), *t*(71) = 4.56, *p* < 0.001, Cohen’s *d* = 0.54, and the incongruent condition (*M* = 3.44, *SD* = 0.45; 95% CI for the difference in means: [0.17, 0.31]), *t*(71) = 6.71, *p* < 0.001, Cohen’s *d* = 0.79, as well as higher intensity ratings in the unimodal compared to the incongruent condition (95% CI for the difference in means: [0.06, 0.18]), *t*(71) = 3.73, *p* = 0.001, Cohen’s *d* = 0.44. Groups did not differ in overall intensity ratings (no main effect of *Group*, *F*(1, 70) = 1.36, *p* = 0.248, *η*^*2*^_*p*_ = .02), or in (in)congruency effects during intensity ratings (no *Group* * *Stimulus Condition* interaction), *F*(1.9, 130.4) = 0.17, *p* = 0.827, *η*^*2*^_*p*_ = 0.002 (see Fig. 2 bottom row left for mean intensity ratings per group and stimulus condition).

Again, the main effect of *Group* was not significant at any of the tested CAPE cut-off values of ≥10 and ≤20 (uncorrected *p* ≥ 0.350, *η*^*2*^_*p*_ ≤ 0.01), but the *Group* * *Stimulus Condition* interaction was significant at a cut-off value of 18 (uncorrected *p* = 0.037, *η*^*2*^_*p*_ = 0.05) due to lower intensity ratings in the high vs. low group particularly in the incongruent condition.

##### Attend voice

A significant main effect of *Stimulus Condition* (*F*(1.9, 131.4) = 86.22, *p* < 0.001, *η*^*2*^_*p*_ = 0.55) revealed higher intensity ratings in the congruent (*M* = 3.60, *SD* = 0.51) compared to both the unimodal (*M* = 3.54, *SD* = 0.48; 95% CI for the difference in means: [0.01, 0.11]), *t*(71) = 2.41, *p* = 0.019, Cohen’s *d* = 0.28, and the incongruent condition (*M* = 3.22, *SD* = 0.43; 95% CI for the difference in means: [0.31, 0.44]), *t*(71) = 10.87, *p* < 0.001, Cohen’s *d* = 1.28, as well as higher intensity ratings in the unimodal compared to the incongruent condition (95% CI for the difference in means: [0.25, 0.37]), *t*(71) = 10.30, *p* < 0.001, Cohen’s *d* = 1.21. Groups did not differ in overall intensity ratings (no main effect of *Group*, *F*(1, 70) = 0.12, *p* = 0.731, *η*^*2*^_*p*_ = 0.002), or in (in)congruency effects during intensity ratings (no *Group* * *Stimulus Condition* interaction), *F*(1.9, 131.4) = 0.23, *p* = 0.783, *η*^*2*^_*p*_ = 0.003 (see Fig. 2 bottom row right for mean intensity ratings per group and stimulus condition).

The main effect of *Group* was not significant at any of the tested CAPE cut-off values of ≥10 and ≤20 (uncorrected *p* ≥ 0.241, *η*^*2*^_*p*_ ≤ 0.02). The *Group* * *Stimulus Condition* interaction was only significant at a cut-off value of 14 (uncorrected *p* = 0.022, *η*^*2*^_*p*_ = 0.06) due to lower intensity ratings in the high (*n* = 10) vs. low (*n* = 62) group particularly in the audiovisual conditions.

### Control tests for attention, processing speed and vigilance

Analysis of TMT data indicated similar completion times (in seconds) in the high compared to the low proneness group both in the TMT-A Version (high proneness: *M* = 30.1 s, *SD* = 10.9; low proneness: *M* = 27.0 s, *SD* = 8.0; 95% CI for the difference in means: [–1.4, 7.6]; *t*(70) = –1.36, *p* = 0.178, Cohen’s *d* = 0.32) and in the TMT-B Version (high proneness: *M* = 54.1 s, *SD* = 13.7; low proneness: *M* = 54.9 s, *SD* = 16.5; 95% CI for the difference in means: [–7.9, 6.3]; *t*(70) = 0.22, *p* = 0.824, Cohen’s *d* = 0.05).

Groups did not differ in overall CPT-IP *d*’-scores (no main effect of *Group*, *F*(1, 70) = 0.36, *p* = 0.550, *η*^*2*^_*p*_ = 0.005), or in CPT-IP *d*’-scores relative to digit load condition (no *Group* * *Digit Load* interaction, *F*(2, 140) = 0.48, *p* = 0.618, *η*^*2*^_*p*_ = 0.007).

### Correlations between experimental data and questionnaires

Pearson correlation coefficients over all participants indicated a negative correlation between the IE difference score congruent-unimodal in the attend face condition and the CAPS (*r* = –0.236, 95% CI [–0.443, –0.004], uncorrected *p* = 0.046), which, however, did not remain significant after Bonferroni-correction. No other correlation coefficients between IE difference scores and CAPS, LSHS or Paranoia Checklist scores reached statistical significance, all *r* < 0.3 and *p* > 0.05 (see Table 2 for Pearson’s *r* values for correlations between IE difference scores, CAPS, LSHS and Paranoia Checklist). Further, no correlation between intensity difference scores and CAPS, LSHS or Paranoia Checklist scores reached statistical significance, all *r* < 0.3 and *p* > 0.05 (see Table 3 for Pearson’s *r* values for correlations between intensity difference scores, CAPS, LSHS and Paranoia Checklist**)**.

**Table 2:** Pearson correlation coefficients between IE difference scores and questionnaire scores.

		1.	2.	3.	4.	5.	6.	7.	8.
Attend Face	1. IE: C-U								
	2. IE: U-I	–0.13							
	3. IE: C-I	0.48***	0.81***						
Attend Voice	4. IE: C-U	0.21	0.11	0.22					
	5. IE: U-I	0.19	0.17	0.27	0.06				
	6. IE: C-I	0.25	0.20	**0.32**	0.44***	0.92***			
	7. CAPS	–0.24	–0.03	–0.17	–0.15	–0.03	–0.08		
	8. LSHS-E	–0.19	0.01	–0.1	–0.18	–0.4	–0.1	0.7***	
	9. PCL^a^	–0.21	0.08	–0.05	0.01	–0.2	–0.18	0.42***	0.52***

*N* = 72. Pearson correlation coefficients *r* > 0.3 are marked in bold. *p*-values are Bonferroni-corrected (see Data Analysis for details). IE Inverse Efficiency; C-U Difference in IE scores between congruent and unimodal conditions; U-I Difference in IE scores between unimodal and incongruent conditions; C-I Difference in IE scores between congruent and incongruent conditions; CAPS total Cardiff Anomalous Perception Scale, sum of endorsed items; LSHS-E Launey-Slade Hallucination Scale-Extended; PCL total Paranoia Checklist, total score.

^a^One participant in the low and one in the high proneness group had ≥50% missings in the Paranoia Checklist. Their Paranoia Checklist scores were corrected by the respective group mean.

***p < 0.001.

**Table 3:** Pearson correlation coefficients *r* between intensity difference scores and questionnaire scores.

		1.	2.	3.	4.	5.	6.	7.	8.
Attend Face	1. INT: C-U								
	2. INT: U-I	–0.27							
	3. INT: C-I	0.5***	0.7***						
Attend Voice	4. INT: C-U	–0.13	0.12	0.01					
	5. INT: U-I	0.02	0.23	0.22	–0.25				
	6. INT: C-I	–0.08	0.30	0.2	0.52***	0.69***			
	7. CAPS	0.18	–0.09	0.05	–0.12	0.05	–0.04		
	8. LSHS-E	0.16	–0.16	–0.02	–0.02	–0.02	–0.03	0.7***	
	9. PCL^a^	0.15	–0.11	0.01	–0.06	–0.05	–0.09	0.42***	52***

*N* = 72. Pearson correlation coefficients *r* > 0.3 are marked in bold. *p*-values are Bonferroni-corrected (see Data Analysis for details). INT Perceived emotional intensity; C-U Difference in perceived intensity between congruent and unimodal conditions; U-I Difference in perceived intensity between unimodal and incongruent conditions; C-I Difference in perceived intensity scores between congruent and incongruent conditions; CAPS total Cardiff Anomalous Perception Scale, sum of endorsed items; LSHS-E Launey-Slade Hallucination Scale-Extended; PCL total Paranoia Checklist, total score.

^a^One participant in the low and one in the high proneness group had ≥50% missings in the Paranoia Checklist. Their Paranoia Checklist scores were corrected by the respective group mean.

***p < 0.001.

## Discussion

We investigated MSI of visual and auditory emotional cues in individuals with high and low psychosis proneness. We adapted the paradigm of Föcker et al.^32^ in which participants had to categorize the emotion of unimodal visual (dynamic faces) and auditory stimuli (voices), as well as crossmodal audio-visual stimuli (either emotionally congruent or incongruent). First, we were able to replicate the main effects of Föcker et al.^32^: Crossmodal emotional categorization was more efficient for congruent audio-visual presentations than for unimodal auditory and visual presentations and least efficient for incongruent audio-visual combinations. Contrary to our hypothesis, we did not find any differences between the high and low psychosis proneness groups, neither for the unimodal nor for the crossmodal conditions. There were also no significant associations between any of the crossmodal (in)congruency effects and self-reported anomalous perceptual experiences, hallucinations or paranoia.

From the lack of a significant difference between individuals with a low vs. high psychosis proneness, we conclude that altered MSI of emotional cues does not seem to be associated with subclinical manifestations of psychosis. In the context of previous studies in clinical samples, which have pointed to altered MSI of emotional cues^16,18–26^, we speculate that altered MSI of emotional cues might emerge later in the developmental trajectory of psychosis and, thus, might be a consequence of deteriorating perception and/or multisensory binding rather than an early risk marker.

Our findings in a psychosis-prone sample deviate from previous studies, indicating impaired unisensory emotion recognition in individuals with a clinically high risk of psychosis^8,9^. Assuming (still) intact multisensory binding processes, multisensory gains might compensate for beginning deficits in unisensory emotion processing. At least partially preserved multisensory gains during emotion processing have in fact been observed in previous clinical studies^16,27,29^ which opens up the possibility that multisensory gains might allow for improving emotion categorization during early phases of psychosis.

It could be speculated that multisensory emotion processing impairments manifest later, while unisensory difficulties are already found in psychosis-prone or clinical high-risk samples^8,9^. Preliminary support for such a progressive decline stems from studies reporting that age, illness duration and symptom severity were correlated with deficient emotion processing in psychosis^61,62^. Previous studies in first-degree relatives of patients with the diagnosis of a psychotic disorder reported deficient facial emotion processing^10^, suggesting that impaired emotion processing might constitute a vulnerability marker for psychosis^8^. Yet, we did not find differences in unisensory emotional categorization in our samples of individuals with low vs. high proneness for psychosis.

Based on the idea that altered MSI of emotional cues might be linked to the experience of perceptual anomalies and perceptual symptoms of psychosis, we tested associations between emotional crossmodal (in)congruency effects and self-reports of anomalous perceptual experiences and psychotic symptoms. The high psychosis proneness group (classified on the basis of overall psychotic liability assessed with the CAPE) reported significantly more perceptual anomalies (assessed with the CAPS) and subclinical hallucinatory experiences (assessed with the LSHS) and showed a trend towards higher paranoid ideation (assessed with the Paranoia Checklist). However, our correlational analyses did not reveal crossmodal (in)congruency effects to be significantly associated with perceptual anomalies, hallucinations or paranoia. To the best of our knowledge, the present study is the first that tried to link experimental results of audio-visual emotional categorization with clinical scales assessing perceptual anomalies of individuals with clinical or subclinical psychosis. The present results are in accord with previous reports which failed to demonstrate correlations between MSI of emotional cues and psychotic symptoms^15,16,23,25,27^. In sum, the results of the present correlational analysis are consistent with the idea that psychotic symptoms are not directly linked to impaired MSI of emotional cues.

It could be argued that our paradigm was not sensitive enough to detect subtle differences in MSI of emotional cues in individuals with a high proneness for psychosis. However, the experimental paradigm adopted from Föcker et al.^32^, which was used in the present study, is highly similar to the experimental paradigms administered in previous studies that had in fact shown deficiencies in MSI of emotional cues in patients with the clinical diagnosis of a psychotic disorder. To our knowledge, however, such an experimental paradigm has not been employed in psychosis proneness samples before. Additionally, the study was powered to detect moderate effects. Thus, we cannot fully exclude the possibility that our design was not sensitive enough to detect potentially very small differences in MSI of emotional cues between high and low psychosis proneness individuals.

Another possibility why we failed to observe the expected group differences is that our high and low psychosis proneness groups might not have been sufficiently distinct from each other. Previous studies on unisensory emotion processing recruited participants who fulfilled established criteria for clinical high-risk^8,9^. By contrast, we recruited participants with high psychosis proneness based on symptom reports in the CAPE, a questionnaire found suitable to quantify psychosis proneness^45,63,64^. Following the threshold procedure by Krkovic et al.^44^, we divided the sample at a score, rather than only including participants with very low and high scores. As a result, the groups might not have been sufficiently distinct to reveal differences in tasks such as the present one assessing MSI of emotional cues, although we did find significant group differences in the questionnaire scores for anomalous perceptual experiences, hallucinations, and, as a trend, for paranoia, suggesting successful grouping as intended based on the CAPE scores. An exploratory analysis indicated that a very high CAPE cut-off value (in most cases of ≥18) might have resulted in a significant group effect in several task conditions, as participants above this value showed higher IE scores and lower intensity rating than participants below this value particularly in the incongruent condition. CAPE scores of ≥18 correspond to the top half of the distribution of clinical psychosis samples^45^, and to the mean score found in an ultra-high-risk sample^64^. The results for the group with CAPE scores of ≥18 suggest that impaired MSI is likely to be more readily identifiable in people with more pronounced symptom profiles. Due to the exploratory nature of this analysis and the small subgroup of participants with a CAPE score ≥18 (*n* = 8), this observation and its interpretation should, however, be treated with caution.

To conclude, we observed indistinguishable MSI of emotional cues in participants with high compared to low psychosis proneness during processing of emotional facial expressions and affective vocal prosody. These findings do not support the notion that altered MSI of emotional cues is associated with psychosis proneness at this low level of severity. Future studies need to investigate at which level of the buildup and further course of psychosis MSI of emotional cues declines.

## Supplementary information

Supplement

## Data Availability

The data that support the findings of this study are available in the research data repository of the University of Hamburg at 10.25592/uhhfdm.17905.

## References

[1] Barkl, S. J., Lah, S., Harris, A. W. F. & Williams, L. M. Facial emotion identification in early-onset and first-episode psychosis: a systematic review with meta-analysis. *Schizophr. Res.* **159**, 62–69 (2014). 25178803 10.1016/j.schres.2014.07.049

[2] Gong, B., Li, Q., Zhao, Y. & Wu, C. Auditory emotion recognition deficits in schizophrenia: a systematic review and meta-analysis. *Asian J. Psychiatry* **65**, 102820 (2021). 10.1016/j.ajp.2021.102820 34482183

[3] Healey, K. M., Bartholomeusz, C. F. & Penn, D. L. Deficits in social cognition in first episode psychosis: a review of the literature. *Clin. Psychol. Rev.* **50**, 108–137 (2016). 27771557 10.1016/j.cpr.2016.10.001

[4] Lin, Y., Ding, H. & Zhang, Y. Emotional prosody processing in schizophrenic patients: a selective review and meta-analysis. *JCM* **7**, 363 (2018). 30336573 10.3390/jcm7100363 PMC6210777

[5] Tseng, H.-H. et al. Facial and prosodic emotion recognition deficits associate with specific clusters of psychotic symptoms in schizophrenia. *PLoS ONE* **8**, e66571 (2013). 23818944 10.1371/journal.pone.0066571 PMC3688591

[6] Brittain, P., Ffytche, D. H., McKendrick, A. & Surguladze, S. Visual processing, social cognition and functional outcome in schizophrenia. *Psychiatry Res.* **178**, 270–275 (2010). 10.1016/j.psychres.2009.09.013 20494457

[7] Pinkham, A. E., Brensinger, C., Kohler, C., Gur, R. E. & Gur, R. C. Actively paranoid patients with schizophrenia over attribute anger to neutral faces. *Schizophr. Res.* **125**, 174–178 (2011). 21112186 10.1016/j.schres.2010.11.006 PMC3031724

[8] Seo, E. et al. Impaired facial emotion recognition in individuals at ultra-high risk for psychosis and associations with schizotypy and paranoia level. *Front. Psychiatry* **11**, 577 (2020). 32676040 10.3389/fpsyt.2020.00577 PMC7333645

[9] van Donkersgoed, R. J. M., Wunderink, L., Nieboer, R., Aleman, A. & Pijnenborg, G. H. M. Social cognition in individuals at ultra-high risk for psychosis: a meta-analysis. *PLoS ONE* **10**, e0141075 (2015). 26510175 10.1371/journal.pone.0141075 PMC4624797

[10] Martin, D. et al. Systematic review and meta-analysis of the relationship between genetic risk for schizophrenia and facial emotion recognition. *Schizophr. Res.* **218**, 7–13 (2020). 31932173 10.1016/j.schres.2019.12.031

[11] Corcoran, C. M. et al. Emotion recognition deficits as predictors of transition in individuals at clinical high risk for schizophrenia: a neurodevelopmental perspective. *Psychol. Med.* **45**, 2959–2973 (2015). 26040537 10.1017/S0033291715000902 PMC5080982

[12] Tseng, H.-H. et al. A systematic review of multisensory cognitive–affective integration in schizophrenia. *Neurosci. Biobehav. Rev.* **55**, 444–452 (2015). 25956248 10.1016/j.neubiorev.2015.04.019

[13] Wallace, M. T., Woynaroski, T. G. & Stevenson, R. A. Multisensory integration as a window into orderly and disrupted cognition and communication. *Annu. Rev. Psychol.* **71**, 193–219 (2020). 31518523 10.1146/annurev-psych-010419-051112

[14] Gröhn, C., Norgren, E. & Eriksson, L. A systematic review of the neural correlates of multisensory integration in schizophrenia. *Schizophr. Res. Cogn.* **27**, 100219 (2022). 34660211 10.1016/j.scog.2021.100219 PMC8502765

[15] Giannitelli, M. et al. Facial, vocal and cross-modal emotion processing in early-onset schizophrenia spectrum disorders. *Schizophr. Res.* **168**, 252–259 (2015). 26297473 10.1016/j.schres.2015.07.039

[16] Mangelinckx, C., Belge, J. B., Maurage, P. & Constant, E. Impaired facial and vocal emotion decoding in schizophrenia is underpinned by basic perceptivo-motor deficits. *Cogn. Neuropsychiatry* **22**, 461–467 (2017). 28974159 10.1080/13546805.2017.1382342

[17] Lin, Y., Ding, H. & Zhang, Y. Multisensory integration of emotion in schizophrenic patients. *Multisens. Res.* **33**, 865–901 (2020). 33706267 10.1163/22134808-bja10016

[18] Castagna, F. et al. Prosody recognition and audiovisual emotion matching in schizophrenia: The contribution of cognition and psychopathology. *Psychiatry Res.* **205**, 192–198 (2013). 22985542 10.1016/j.psychres.2012.08.038

[19] de Gelder, B. et al. Multisensory integration of emotional faces and voices in schizophrenics. *Schizophr. Res.* **72**, 195–203 (2005). 15560964 10.1016/j.schres.2004.02.013

[20] de Jong, J. J., Hodiamont, P. P. G., Van den Stock, J. & de Gelder, B. Audiovisual emotion recognition in schizophrenia: reduced integration of facial and vocal affect. *Schizophr. Res.* **107**, 286–293 (2009). 18986799 10.1016/j.schres.2008.10.001

[21] de Jong, J. J., Hodiamont, P. P. G. & de Gelder, B. Modality-specific attention and multisensory integration of emotions in schizophrenia: reduced regulatory effects. *Schizophr. Res.* **122**, 136–143 (2010). 20554159 10.1016/j.schres.2010.04.010

[22] Fiszdon, J. M. & Bell, M. D. Effects of presentation modality and valence on affect recognition performance in schizophrenia and healthy controls. *Psychiatry Res.* **170**, 114–118 (2009). 19900721 10.1016/j.psychres.2008.11.014

[23] Jeong, J. W., Kim, H. T., Lee, S.-H. & Lee, H. Effects of an audiovisual emotion perception training for schizophrenia: a preliminary study. *Front. Psychiatry* **12**, 522094 (2021). 34025462 10.3389/fpsyt.2021.522094 PMC8131526

[24] Portnova, G., Maslennikova, A., Zakharova, N. & Martynova, O. The deficit of multimodal perception of congruent and non-congruent fearful expressions in patients with schizophrenia: the ERP study. *Brain Sci.* **11**, 96 (2021). 33451054 10.3390/brainsci11010096 PMC7828540

[25] Seubert, J. Multisensory integration of emotionally valenced olfactory–visual information in patients with schizophrenia and healthy controls. *J. Psychiatry Neurosci.* **35**, 185–194 (2010). 20420769 10.1503/jpn.090094 PMC2861135

[26] Van den Stock, J., de Jong, S. J., Hodiamont, P. P. G. & de Gelder, B. Perceiving emotions from bodily expressions and multisensory integration of emotion cues in schizophrenia. *Soc. Neurosci.* **6**, 537–547 (2011). 21777157 10.1080/17470919.2011.568790

[27] Müller, V. I., Kellermann, T. S., Seligman, S. C., Turetsky, B. I. & Eickhoff, S. B. Modulation of affective face processing deficits in Schizophrenia by congruent emotional sounds. *Soc. Cogn. Affect Neurosci.* **9**, 436–444 (2014). 22977201 10.1093/scan/nss107 PMC3989119

[28] Sestito, M. et al. Facial reactions in response to dynamic emotional stimuli in different modalities in patients suffering from schizophrenia: a behavioral and EMG study. *Front. Hum. Neurosci*. **7**, 368 (2013). 10.3389/fnhum.2013.00368 PMC3719033 23888132

[29] Simpson, C., Pinkham, A. E., Kelsven, S. & Sasson, N. J. Emotion recognition abilities across stimulus modalities in schizophrenia and the role of visual attention. *Schizophr. Res.* **151**, 102–106 (2013). 24126043 10.1016/j.schres.2013.09.026

[30] Zvyagintsev, M., Parisi, C., Chechko, N., Nikolaev, A. R. & Mathiak, K. Attention and multisensory integration of emotions in schizophrenia. *Front. Hum. Neurosci.* **7**, 674 (2013). 24151459 10.3389/fnhum.2013.00674 PMC3798810

[31] Collignon, O. et al. Audio-visual integration of emotion expression. *Brain Res.* **1242**, 126–135 (2008). 18495094 10.1016/j.brainres.2008.04.023

[32] Föcker, J., Gondan, M. & Röder, B. Preattentive processing of audio-visual emotional signals. *Acta Psychol.* **137**, 36–47 (2011). 10.1016/j.actpsy.2011.02.004 21397889

[33] Opoku-Baah, C. et al. Visual influences on auditory behavioral, neural, and perceptual processes: a review. *JARO* **22***,* 365–386 (2021). 10.1007/s10162-021-00789-0 PMC8329114 34014416

[34] Postmes, L. et al. Schizophrenia as a self-disorder due to perceptual incoherence. *Schizophr. Res.* **152**, 41–50 (2014). 23973319 10.1016/j.schres.2013.07.027

[35] Thakkar, K. N. & Rolfs, M. Disrupted corollary discharge in schizophrenia: evidence from the oculomotor system. *Biol. Psychiatry Cogn. Neurosci. Neuroimaging* **4**, 773–781 (2019). 31105039 10.1016/j.bpsc.2019.03.009 PMC6733648

[36] Bell, V., Halligan, P. W. & Ellis, H. D. The Cardiff Anomalous Perceptions Scale (CAPS): a new validated measure of anomalous perceptual experience. *Schizophr. Bull.* **32**, 366–377 (2006). 16237200 10.1093/schbul/sbj014 PMC2632213

[37] Garety, P. A., Kuipers, E., Fowler, D., Freeman, D. & Bebbington, P. E. A cognitive model of the positive symptoms of psychosis. *Psychol. Med.* **31**, 189–195 (2001). 11232907 10.1017/s0033291701003312

[38] Denecke, S., Schönig, S. N., Bott, A., Faße, J. L. & Lincoln, T. M. Bridging perspectives - A review and synthesis of 53 theoretical models of delusions. *Clin. Psychol. Rev.* **114**, 102510 (2024). 39515077 10.1016/j.cpr.2024.102510

[39] Addington, J. et al. Substance use in clinical high risk for psychosis: a review of the literature. *Early Interv. Psychiatry* **8**, 104–112 (2014). 10.1111/eip.12100 PMC4356483 24224849

[40] McAusland, L. et al. Anxiety in youth at clinical high risk for psychosis. *Early Interv. Psychiatry* **11**, 480–487 (2017). 10.1111/eip.12274 PMC4912451 26456932

[41] Verdoux, H. et al. Increased occurrence of depression in psychosis-prone subjects: a follow-up study in primary care settings. *Compr. Psychiatry* **40**, 462–468 (1999). 10579379 10.1016/s0010-440x(99)90091-3

[42] Faul, F., Erdfelder, E., Lang, A.-G. & Buchner, A. G*Power 3: A flexible statistical power analysis program for the social, behavioral, and biomedical sciences. *Behav. Res. Methods* **39**, 175–191 (2007). 10.3758/bf03193146 17695343

[43] Stefanis, N. C. et al. Evidence that three dimensions of psychosis have a distribution in the general population. *Psychol. Med.* **32**, 347–358 (2002). 11866327 10.1017/s0033291701005141

[44] Krkovic, K., Clamor, A., Schlier, B. & Lincoln, T. M. Emotions and persecutory ideation in daily life: on the trail of the “chicken and egg” problem. *J. Abnorm. Psychol.* **129**, 215–223 (2020). 31829637 10.1037/abn0000495

[45] Jaya, E. S. et al. The community assessment of psychic experiences: optimal cut-off scores for detecting individuals with a psychotic disorder. *Int. J. Methods Psychiatr. Res.* **30**, e1893 (2021). 34464487 10.1002/mpr.1893 PMC8633944

[46] Schlier, B., Jaya, E. S., Moritz, S. & Lincoln, T. M. The community assessment of psychic experiences measures nine clusters of psychosis-like experiences: A validation of the German version of the CAPE. *Schizophr. Res.* **169**, 274–279 (2015). 26545299 10.1016/j.schres.2015.10.034

[47] Dong, F. et al. Cognitive deficits profiles in the first-episode of schizophrenia, clinical high risk of psychosis, and genetically high-risk of psychosis. *Front. Psychiatry* **14**, 1292141 (2023). 38146278 10.3389/fpsyt.2023.1292141 PMC10749319

[48] Melillo, A. et al. Correlations between negative symptoms and cognitive deficits in individuals at first psychotic episode or at high risk of psychosis: a systematic review. *JCM* **12**, 7095 (2023). 38002707 10.3390/jcm12227095 PMC10672428

[49] Reitan, R. M. & Wolfson, D. *The Halstead-Reitan Neuropsychological Test Battery: Theory and Interpretation*. (Neuropsychology Press, Tucson, AZ, 1985).

[50] Nuechterlein, K. H. et al. The MATRICS consensus cognitive battery, part 1: test selection, reliability, and validity. *AJP* **165**, 203–213 (2008). 10.1176/appi.ajp.2007.07010042 18172019

[51] Bentall, R. P. & Slade, P. D. Reliability of a scale measuring disposition towards hallucination: a brief report. *Personal. Individ. Diff.* **6**, 527–529 (1985).

[52] Siddi, S. et al. A cross-national investigation of hallucination-like experiences in 10 countries: the E-CLECTIC study. *Schizophr. Bull.* **45**, S43–S55 (2019). 30715543 10.1093/schbul/sby156 PMC6357978

[53] Freeman, D. et al. Psychological investigation of the structure of paranoia in a non-clinical population. *Br. J. Psychiatry* **186**, 427–435 (2005). 15863749 10.1192/bjp.186.5.427

[54] Fowler, D. et al. The Brief Core Schema Scales (BCSS): psychometric properties and associations with paranoia and grandiosity in non-clinical and psychosis samples. *Psychol. Med.* **36**, 749–759 (2006). 16563204 10.1017/S0033291706007355

[55] Townsend, J. T. & Ashby, F. G. Methods of Modeling Capacity in Simple Processing Systems. in *Cognitive Theory* 199–239 (Erlbaum, Hillsdale, NJ, 1978).

[56] Huynh, H. & Feldt, L. S. Estimation of the box correction for degrees of freedom from sample data in randomized block and split-plot designs. *J. Educ. Stat.* **1**, 69–82 (1976).

[57] Edgell, S. E. & Noon, S. M. Effect of violation of normality on the t test of the correlation coefficient. *Psychol. Bull.* **95**, 576–583 (1984).

[58] Havlicek, L. L. & Peterson, N. L. Effect of the violation of assumptions upon significance levels of the Pearson r. *Psychol. Bull.* **84**, 373–377 (1977).

[59] Ventura-León, J., Peña-Calero, B. N. & Burga-León, A. The effect of normality and outliers on bivariate correlation coefficients in psychology: A Monte Carlo simulation. *J. Gen. Psychol.* **150**, 405–422 (2023). 35792742 10.1080/00221309.2022.2094310

[60] Cornblatt, B. A., Risch, N. J., Faris, G., Friedman, D. & Erlenmeyer-Kimling, L. The Continuous Performance Test, Identical Pairs version (CPT-IP): I. New findings about sustained attention in normal families. *Psychiatry Res.* **26**, 223–238 (1988). 3237915 10.1016/0165-1781(88)90076-5

[61] Edwards, J., Pattison, P. E., Jackson, H. J. & Wales, R. J. Facial affect and affective prosody recognition in first-episode schizophrenia. *Schizophr. Res.* **48**, 235–253 (2001). 11295377 10.1016/s0920-9964(00)00099-2

[62] Kohler, C. G., Walker, J. B., Martin, E. A., Healey, K. M. & Moberg, P. J. Facial emotion perception in schizophrenia: a meta-analytic review. *Schizophr. Bull.* **36**, 1009–1019 (2010). 19329561 10.1093/schbul/sbn192 PMC2930336

[63] Bukenaite, A. et al. Usefulness of the CAPE-P15 for detecting people at ultra-high risk for psychosis: psychometric properties and cut-off values. *Schizophr. Res.* **189**, 69–74 (2017). 28254243 10.1016/j.schres.2017.02.017

[64] Mossaheb, N. et al. The Community Assessment of Psychic Experience (CAPE) questionnaire as a screening-instrument in the detection of individuals at ultra-high risk for psychosis. *Schizophr. Res.* **141**, 210–214 (2012). 22986044 10.1016/j.schres.2012.08.008

