# Supplementary material for: Multisensory integration of affective faces and voices in psychosis proneness
Source: Schizophrenia (Heidelb). 2025 Sep 22;11(1):118. doi: 10.1038/s41537-025-00676-0 (PMC12454648; doi:10.1038/s41537-025-00676-0)
Supplement: Supplementary file 1 — Supplement [file 41537_2025_676_MOESM1_ESM.pdf]

Supplement

Multisensory integration of affective faces and voices in psychosis proneness

Authors: Andreas Weiss; Patrick Bruns; Brigitte Röder; Tania M. Lincoln

1 Participant Characteristics of Anxiety, Depression, and Substance Abuse

Since previous evidence indicated a higher prevalence of anxiety, depression, and substance abuse in psychosis proneness [1-3], we did not exclude currently present, mild forms of said diagnoses in order to recruit a representative sample with psychosis proneness. Participants answered the following questions via self-report: "Have you ever been diagnosed with a mental disorder? If yes, which? Is it currently present or was it present in the last six months? Is/was it a severe case?"

Relative to total sample size, positive responses were few and similarly distributed over both groups. CAPE positive scores of participants with diagnosis ($CAPE_{pos}$ scores in high proneness group: 11, 14, 12, 9; $CAPE_{pos}$ scores in low proneness group: 8, 5, 1, 4, 1) were relatively distributed and did not fall in upper extreme values. Thus, it can be argued that the experimental results were not significantly confounded by anxiety, depression or substance abuse (see Supplementary Table S1 for number of cases for each disorder).

Table S1

Number, current status and severity of cases for anxiety, depression, and substance per group

Diagnosis	n_{total} high	n_{total} low	$n_{current}$ high	$n_{current}$ low	$n_{6months}$ high	$n_{6months}$ low
Anxiety	3	3	1 (mild)	1 (mild)	1 (severe)	1 (mild)
Depression	5	4	0	0	1 (severe)	0
Substance Abuse	0	0	0	0	0	0

Notes. $N = 72$. n_{total} = total number of reported cases; $n_{current}$ = number of currently present cases; $n_{6months}$ = number of cases present in the last 6 months.

Severity of disorder is written in parentheses behind the case number in the columns for $n_{current}$ and $n_{6months}$.

2 Analyses with Attention as Additional Within-Factor

To check for potential attention-specific group differences, we calculated exploratory mixed ANOVAs with the between-factor *Group* (low proneness vs high proneness) and the within-factors *Stimulus Condition* (unimodal vs bimodal congruent vs bimodal incongruent) and *Attention* (attend face vs attend voice) on IE scores and intensity ratings.

2.1 Inverse Efficiency

A significant main effect of *Stimulus Condition*, $F(1.3, 93.6) = 121.\ 2$, $p < .001$, $\eta^2_p = .63$, revealed lower IE-scores in congruent ($M = 1790$, SD = 332) compared to both the unimodal ($M = 1941$, $SD = 345$; 95% CI for the difference in means: [-186, -114]), $t(71) = -8.34$, $p < .001$, Cohen's $d = 0.98$, and the incongruent condition ($M = 2274$, $SD = 443$; 95% CI for the difference in means: [-563, -405]), $t(71) = -12.22$, $p < .001$, Cohen's $d = 1.44$, as well as lower IE scores in the unimodal compared to the incongruent condition (95% CI for the difference in means: [-400, -267], $t(71) = -10.01$, $p < 001$, Cohen's $d = 1.18$. IE-scores were lower in attend face blocks ($M = 1920$, $SD = 363$) compared to attend voice blocks ($M = 2083$, $SD = 361$; 95% CI for the difference in means: [-217, -109]; main effect of *Attention*, $F(1, 70) = 38.50$, $p < .001$, $\eta^2_p = .36$). Further, there were significant interactions between *Group* * *Attention*, $F(1, 70) = 5.26$, $p = .025$, $\eta^2_p = .07$, and *Stimulus Condition* * *Attention*, $F(1.4, 101.3) = 30.92$, $p < .001$, $\eta^2_p = .31$. There was no main effect of *Group*, $F(1, 70) = 0.48$, $p = .490$, $\eta^2_p = .007$, and no *Group* * *Stimulus Condition*, $F(1.3, 93.6) = 0.26$, $p = .677$, $\eta^2_p = .004$, or *Group* * *Stimulus Condition* * *Attention* interaction, $F(1.4, 101.3) = 0.49$, $p = .554$, $\eta^2_p = .007$.

Attention-specific post-hoc tests for group comparisons revealed similar IE scores in the high proneness ($M = 1862$, $SD = 324$) compared to the low proneness group ($M = 1979$, $SD = 395$; 95% CI for the difference in means: [-286, 53]) in the attend face condition, $t(70) = 1.37$, $p = .175$, Cohen's $d = 0.32$, and in the attend voice condition (high proneness: $M = 2085$, $SD = 375$;

low proneness: *M* = 2081, *SD* = 351; 95% CI for the difference in means: [-167, 175]), *t*(70) = -0.46, *p* = .964, Cohen's *d* = 0.01. Condition-specific post-hoc tests for attention condition comparisons revealed lower IE scores in the attend face compared to the attend voice condition in incongruent trials (face: *M* = 2082, *SD* = 457; voice: *M* = 2467, SD = 544; 95% CI for the difference in means: [-496, -274]) *t*(71) = -6.91, *p* < .001, Cohen's *d* = 0.81, and in unimodal trials (face: *M* = 1898, *SD* = 396; voice: *M* = 1983, *SD* = 327; 95% CI for the difference in means: [-139, -32]), *t*(71) = -3.18, *p* = .002, Cohen's *d* = 0.38, but not in congruent trials (face: *M* = 1781, *SD* = 349; voice: *M* = 1800, *SD* = 358; 95% CI for the difference in means: [-76, 37]), *t*(71) = -0.68, *p* = .499, Cohens's *d* = 0.08.

2.2 Intensity Ratings

Analogue to the IE analysis, we calculated an exploratory 2 (*Group*) x 3 (*Stimulus Condition*) x 2 (*Attention*) rm-ANOVA for intensity scores. A significant main effect of *Stimulus Condition* (*F*(1.7, 117.5) = 90.18, *p* < .001, η^2_p = .56) revealed higher intensity ratings in the congruent condition (*M* = 3.64, *SD* = 0.47) compared to both the unimodal (*M* = 3.55, *SD* = 0.42; 95% CI for the difference in means: [0.06, 0.12]), *t*(71) = 5.30, *p* < .001, Cohen's *d* = 0.63, and the incongruent condition (*M* = 3.33, *SD* = 0.41; 95% CI for the difference in means: [0.25, 0.36]), *t*(71) = 11.29, *p* < .001, Cohen's *d* = 1.33, as well as higher intensity ratings in the unimodal compared to the incongruent condition (95% CI for the difference in means: [0.17, 0.26], *t*(71) = 8.84, *p* <.001, Cohen's *d* = 1.04. Intensity ratings were higher in attend face blocks (*M* = 3.56, *SD* = 0.41) compared to attend voice blocks (*M* = 3.45, *SD* = 0.45; main effect of *Attention*, *F*(1, 70) = 20.47, *p* < .001 , η^2_p = .23). Further, there was a significant *Stimulus Condition* * *Attention* interaction, *F*(2, 140) = 11.88, *p* < .001 , η^2_p = .15. There was no main effect of *Group*, *F*(1, 70) = 0.57, *p* = .451, η^2_p = .008, no *Group* * *Stimulus Condition*, *F*(2, 140)

= 0.03, p = .971, η^2_p < .001, no *Group * Attention*, F(1, 70) = 2.57, p = .113, η^2_p = .04, and no *Group * Stimulus Condition * Attention* interaction, F(2, 140) = 0.42, p = .657, η^2_p = .006.

Condition-specific post-hoc tests revealed higher intensity ratings in the attend face compared to the attend voice condition in congruent (face: M = 3.68, SD = 0.46; voice: M = 3.60, SD = 0.51; 95% CI for the difference in means: [0.02, 0.14]), t(71) = 2.60, p = .011, Cohen's d = 0.31, and incongruent trials (face: M = 3.44, SD = 0.45; voice: M = 3.22, SD = 0.43; 95% CI for the difference in means: [0.15, 0.29]), t(71) = 6.16, p < .001, Cohen's d = 0.73, but not in unimodal trials (face: M = 3.56, SD = 0.41; voice: M = 3.53, SD = 0.48; 95% CI for the difference in means: [-0.04, 0.09]), t(71) = 0.72, p = .475, Cohen's d = 0.09.

3 Analyses of Reaction Times and Accuracy

3.1 Reaction Times

Attend Face. A significant main effect of *Stimulus Condition* (F(2, 140) = 4.15, p = .018, η^2_p = 0.06) revealed lower reaction times in the congruent (M = 1568, SD = 277) compared to the incongruent condition (M = 1608, SD = 256; 95% CI for the difference in means: [-67, -12]), t(71) = -2.90, p = .005, Cohen's d = 0.34, but no differences between the unimodal (M = 1582, SD = 280) and both the congruent (95% CI for the difference in means: [-40, 13]), t(71) = -1.04, p = .300, Cohen's d = 0.12, and incongruent condition (95% CI for the difference in means: [-56, 4]), t(71) = -1.74, p = .086, Cohen's d = 0.21. Groups did not differ in overall RT (no main effect of *Group*, F(1, 70) = 1.83, p = .180, η^2_p = 0.03) or in in/-congruency effects on RT (no *Group * Stimulus Condition* interaction, F(2, 140) = 0.45, p = .636, η^2_p = 0.006; see Supplementary Figure S1 top row left for mean RT per group and stimulus condition).

Attend Voice. A significant main effect of *Stimulus Condition* ($F(2, 140) = 47.65$, $p < .001$, $\eta^2_p = 0.41$) revealed lower reactions times in the congruent ($M = 1622$, $SD = 277$) compared to the incongruent condition ($M = 1753$, $SD = 261$; 95% CI for the difference in means: [-160, -101]), $t(71) = -8.78$, $p < .001$, Cohen's $d = 1.03$, and lower reactions times in the unimodal ($M = 1633$, $SD = 247$) compared to the incongruent condition (95% CI for the difference in means: [-149, -91], $t(71) = -8.28$, $p < .001$, Cohen's $d = 0.98$, but no differences between the congruent and the unimodal condition (95% CI for the difference in means: [-42, 20]), $t(71) = -0.69$, $p = .490$, Cohen's $d = 0.08$. Groups did not differ in overall RT (no main effect of *Group*, $F(1, 70) = 0.52$, $p = .475$, $\eta^2_p = 0.007$) or in in/-congruency effects on RT (no *Group * Stimulus Condition* interaction, $F(2, 140) = 1.95$, $p = .146$, $\eta^2_p = 0.03$; see Supplementary Figure S1 top row right for mean RT per group and stimulus condition).

3.2 Accuracy

Attend Face. A 2 (*Group*) x 3 (*Stimulus Condition*) mixed ANOVA on accuracy in attend face blocks was not interpretable due to lack of homogeneity of covariances (Box's test: $p = .035$) and lack of homogeneity of error variances (Levene's test for unimodal trials: $p = .032$). Therefore, we report t-tests for the factors *Group* and *Stimulus Condition*, as recommended [4]. Accuracy scores were higher in the congruent ($M = 88.6$, $SD = 6.6$) compared to both the unimodal ($M = 84.0$, $SD = 6.5$; 95% CI for the difference in means: [2.8, 6.3]), $t(71) = 5.28$, $p < .001$, Cohen's $d = 0.62$, and the incongruent condition ($M = 78.7$, $SD = 10.4$; 95% CI for the difference in means: [7.4, 12.3]), $t(71) = 8.05$, $p < .001$, Cohen's $d = 0.95$, as well as higher in the unimodal compared to the incongruent condition (95% CI for the difference in means: [3.1, 7.5], $t(71) = 4.71$, $p < .001$, Cohen's $d = 0.56$. There was no difference in accuracy between the high proneness ($M = 84.1$, $SD = 6.6$) and the low proneness group ($M = 83.4$, $SD = 5.5$; 95% CI for the

difference in means: [-2.2, 3.5]), *t*(70) = -0.46, *p* = .645, Cohen's *d* = 0.11; see Supplementary Figure S1 bottom row left for accuracy scores per group and stimulus condition).

Attend Voice. A 2 (*Group*) x 3 (*Stimulus Condition*) mixed ANOVA for accuracy in attend voice blocks was not interpretable due to lack of homogeneity of covariances (Box's test: *p* = .036) and to a lack of homogeneity of error variances (Levene's test for congruent trials: *p* = .029). Therefore, we will report t-Tests for the factors *Group* and *Stimulus Condition*, as recommended [4]. Accuracy scores were higher in the congruent (*M* = 90.77, *SD* = 6.16) compared to both the unimodal (*M* = 82.74, *SD* = 6.15), *t*(71) = 11.58, *p* < .001, and the incongruent condition (*M* = 72.78, *SD* = 11.21), *t*(71) = 14.20, *p* < .001, as well as higher in the unimodal compared to the incongruent condition, *t*(71) = 9.08, *p* < .001. There was no difference in accuracy between the high proneness (*M* = 81.2, *SD* = 7.2) and the low proneness group (*M* = 83.0, *SD* = 5.4; 95% CI for the difference in means: [-4.9, 1.1]), *t*(70) = 1.26, *p* = .212, Cohen's *d* = 0.30 (see Supplementary Figure S1 bottom row right for accuracy scores per group and stimulus condition).

Supplementary Figure S1

RT and Accuracy per Group and Stimulus Condition, Separated by Attention Condition

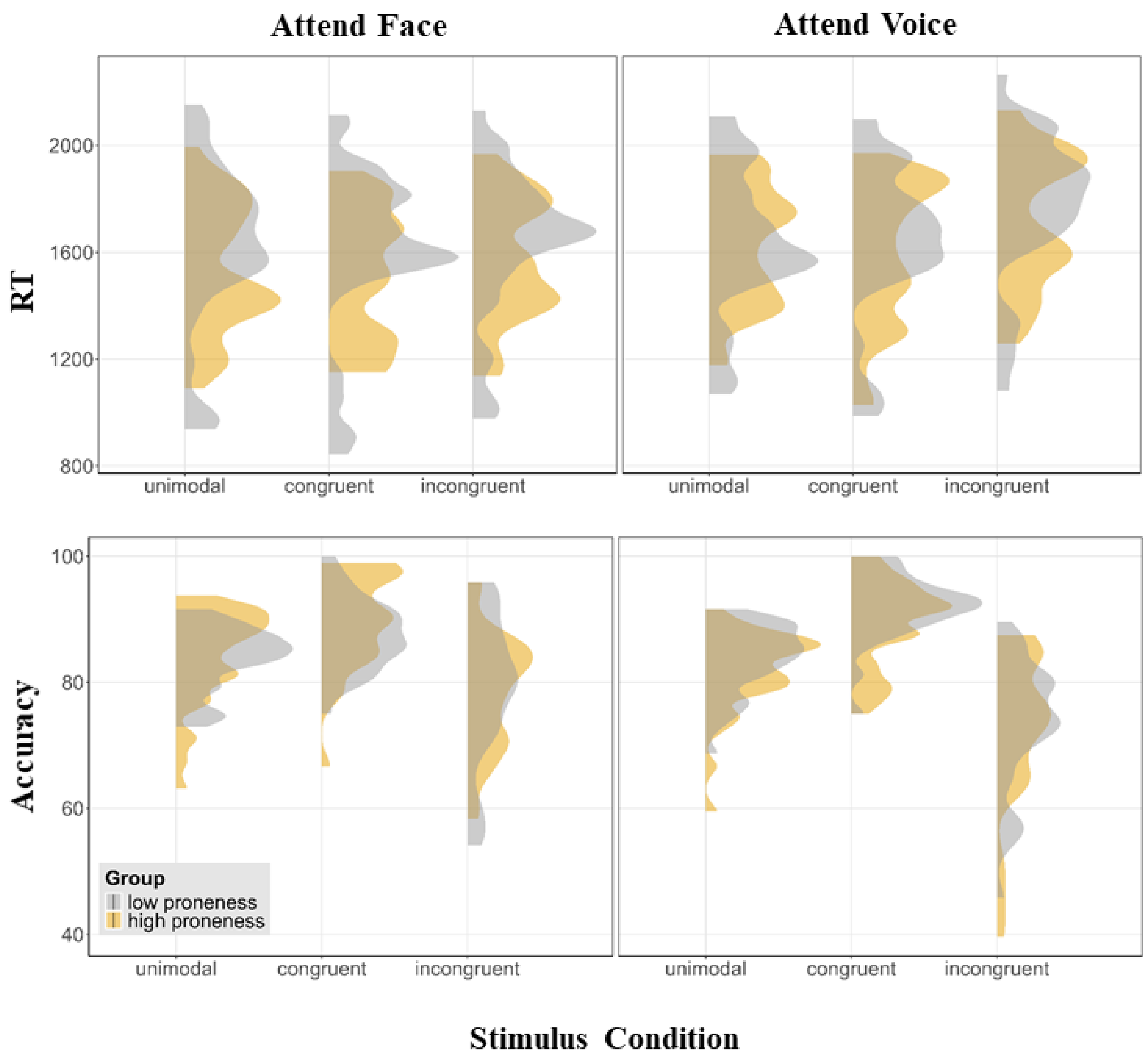

Notes. n_{low} = 36, n_{high} = 36. For both groups, the distributions of reaction times and accuracy are depicted per stimulus condition and attention condition. Congruent = bimodal emotionally congruent; incongruent = bimodal emotionally incongruent.

4 Exploratory Emotion-Specific Analysis

4.1 Methods

We explored if both groups differed in emotion recognition performance regarding the target emotion. Therefore, we additionally calculated mean RT, accuracy and mean perceived intensity separately for each of the four target emotions per participant. IE scores could not be calculated due to low accuracy scores in some target emotion * stimulus condition * attention condition combinations. We observed that calculating IE scores would result in very high extreme values and violations of analysis requirements. Mean RT, accuracy and mean perceived intensity were exploratory analyzed by means of mixed ANOVAs with the factors *Group* and *Stimulus Condition*, separately for each target emotion and attention condition, followed by Bonferroni-corrected post-hoc t-tests. Post-hoc comparisons for the within-factor *Stimulus Condition* are reported between congruent and unimodal trials, regarding a possible congruency facilitation effect (lower RT, higher accuracy, and higher perceived emotional intensity in congruent compared to unimodal trials), and between incongruent and unimodal trials, regarding a possible incongruency interference effect (higher RT, lower accuracy, and lower perceived emotional intensity in incongruent compared to unimodal trials). Comparisons between congruent and incongruent trials are not informative for the research question and are hence not reported.

4.2 Results

Target Emotion Happy. Separate *Group* * *Stimulus Condition* mixed ANOVAs in attend face trials with target emotion happy revealed neither a main effect of *Group*, $F(1, 70) = 0.93$, $p = .338$, $\eta^2_p = 0.01$, nor a main effect of *Stimulus Condition*, $F(2, 140) = 1.79$, $p = .171$, $\eta^2_p = 0.03$, on mean RT. There was a non-significant trend for a *Group* * *Stimulus Condition* interaction on mean RT in attend face trials, $F(2, 140) = 2.97$, $p = .055$, $\eta^2_p = 0.04$. Post-hoc comparisons revealed a non-significant trend for lower mean RT in the high proneness compared to the low proneness group in congruent happy face trials, $t(70) = -1.83$, $p = .072$, Cohen’s $d = -0.43$, but not in unimodal, $t(70) = -0.67$, $p = .505$, Cohen’s $d = -0.16$, or incongruent trials, $t(70) = -0.29$, $p = .775$, Cohen’s $d = -0.07$. The high proneness group showed a congruency facilitation effect, $t(35) = -2.22$, $p = .033$, Cohen’s $d = -0.37$, but no incongruency interference effect $t(35) = 1.11$, $p = .275$, Cohen’s $d = 0.19$, on mean RT in happy face stimuli, whereas the low proneness group neither showed a congruency facilitation, $t(35) = 0.41$, $p = .688$, Cohen’s $d = 0.07$, nor an incongruency interference effect, $t(35) = 0.12$, $p = .907$, Cohen’s $d = 0.02$. There was neither a main effect of *Group*, $F(1, 70) = 2.06$, $p =$

.156, $\eta^2_p = 0.03$, nor a *Group * Stimulus Condition* interaction, $F(1.7, 121.7) = 0.99$, $p = .366$, $\eta^2_p = 0.01$, on accuracy in attend face trials. The factor *Stimulus Condition* showed a non-significant trend for a main effect on accuracy in attend face trials, $F(1.7, 121.7) = 2.75$, $p = .075$, $\eta^2_p = 0.04$, revealing a congruency facilitation effect on accuracy in happy face stimuli, $t(71) = 2.72$, $p = .022$, Cohen's $d = 0.32$. Further, there was no main effect of *Group*, $F(1, 70) = 1.04$, $p = .312$, $\eta^2_p = 0.02$, and no *Group * Stimulus Condition* interaction on mean perceived intensity in attend face trials, $F(2, 140) = 0.6$, $p = .549$, $\eta^2_p = 0.009$. There was a significant main effect of *Stimulus Condition* on mean perceived intensity in attend face trials, $F(2, 140) = 5.46$, $p = .005$, $\eta^2_p = 0.07$, indicating a non-significant trend for a congruency facilitation effect when rating happy face stimuli, $t(71) = 2.30$, $p = .073$, Cohen's $d = 0.27$.

Separate *Group * Stimulus Condition* mixed ANOVAs in attend voice trials with target emotion happy revealed no main effect of *Group* on mean RT, $F(1, 68) = 0.51$, $p = .479$, $\eta^2_p = 0.007$. There was a highly significant main effect of *Stimulus Condition*, $F(2, 136) = 42.31$, $p < .001$, $\eta^2_p = 0.38$, and a non-significant trend for a *Group * Stimulus Condition* interaction, $F(2, 136) = 3$, $p = .053$, $\eta^2_p = 0.04$, on mean RT in attend voice trials. Post-hoc comparisons revealed similar mean RT for both groups in unimodal, $t(70) = -0.1$, $p = .919$, Cohen's $d = -0.02$, congruent, $t(70) = -0.75$, $p = .454$, Cohen's $d = -0.18$, and incongruent happy voice trials, $t(68) = .1{,}61$, $p = .113$, Cohen's $d = -0.38$. The high proneness group showed both a congruency facilitation, $t(35) = -2.29$, $p = .029$, Cohen's $d = -0.38$, and an incongruency interference effect, $t(34) = 2.63$, $p = .013$, Cohen's $d = 0.44$, on mean RT in happy voice trials, whereas the low proneness group showed an incongruency interference, $t(34) = 6.37$, $p < .001$, Cohen's $d = 1.08$, but no congruency facilitation effect, $t(35) = -0.92$, $p = .363$, Cohen's $d = -0.15$. There was no main effect of *Group* on accuracy in attend voice trials, $F(1, 70) = 0.21$, $p = .645$, $\eta^2_p = 0.003$. There was a highly significant main effect of *Stimulus Condition*, $F(1.6, 114.3) = 153.04$, $p < .001$, $\eta^2_p = 0.69$, and a non-significant trend for a *Group * Stimulus Condition* interaction, $F(1.6, 114.3) = 2.54$, $p = .094$, $\eta^2_p = 0.04$, on accuracy in attend voice trials. Post-hoc comparisons revealed similar accuracy for both groups in unimodal, $t(70) = -1.0$, $p = .321$, Cohen's $d = -0.24$, congruent, $t(70) = 0.74$, $p = .465$, Cohen's $d = 0.17$, and incongruent happy voice trials, $t(70) = 1.13$, $p = .264$, Cohen's $d = 0.27$. The high proneness group showed both a congruency facilitation, $t(35) = 7.61$, $p < .001$, Cohen's $d = 1.27$, and incongruency interference effect, $t(35) = -5.49$, $p < .001$, Cohen's $d = 0.92$, on accuracy in happy voice trials. Both a congruency facilitation, $t(35) = 4.85$, $p < .001$, Cohen's $d = 0.81$, and an incongruency interference effect,

$t(35) = -9.57$, $p < .001$, Cohen's $d = -1.59$, on accuracy in happy voice trials could also be observed in the low proneness group. There was no main effect of *Group*, $F(1, 70) = 0.15$, $p = .703$, $\eta^2_p = 0.002$, and no *Group * Stimulus Condition* interaction, $F(2, 140) = 0.2$, $p = .819$, $\eta^2_p = 0.003$, on mean perceived intensity in attend voice trials. The main effect of *Stimulus Condition* on mean perceived intensity in attend voice trials was highly significant, $F(2, 140) = 54.95$, $p < .001$, $\eta^2_p = 0.44$, revealing both a congruency facilitation effect, $t(71) = 5.54$, $p < .001$, Cohen's $d = 0.65$, and an incongruency interference effect, $t(71) = -5.62$, $p < .001$, Cohen's $d = -.066$, when rating happy face stimuli.

In sum, both groups overall showed similar mean RT, accuracy and mean intensity ratings in both attend face and voice trials with target emotion happy. Overall, congruency facilitation and/or incongruency interference effects could be found for all measures in trials with target emotion happy in both attention conditions except for mean RT in attend face trials. In attend face trials, the high proneness group showed both a congruency facilitation and an incongruency interference effect on mean RT, whereas the low proneness group showed neither effect. Apart from this, both groups showed a comparable pattern of in-/congruency effects for all measures in happy face and voice trials (see Supplementary Figure S2 top row for mean RT, accuracy and mean perceived intensity for target emotion happy).

Target Emotion Angry. Separate *Group * Stimulus Condition* mixed ANOVAs in attend face trials with target emotion angry revealed neither a main effect of *Group*, $F(1, 70) = 1.7$, $p = .196$, $\eta^2_p = 0.02$, nor of *Stimulus Condition*, $F(2, 140) = 2.30$, $p = .104$, $\eta^2_p = 0.03$, and no *Group * Stimulus Condition* interaction, $F(2, 140) = 0.77$, $p = .464$, $\eta^2_p = 0.01$, on mean RT. There was no main effect of *Group* on accuracy in attend face trials, $F(1, 70) = 1.26$, $p = .265$, $\eta^2_p = 0.02$, and no *Group * Stimulus Condition* interaction, $F(1.6, 110.7) = 0.95$, $p = .372$, $\eta^2_p = 0.01$ (note: there was no homogeneity of covariances in accuracy in angry face trials, Box-test $p = .001$, prohibiting an interpretation of the interaction). The main effect of *Stimulus Condition* on accuracy in attend face trials was highly significant, $F(1.6, 110.7) = 14.52$, $p < .001$, $\eta^2_p = 0.17$, indicating an incongruency interference effect on accuracy in angry face stimuli, $t(71) = -4$, $p < .001$, Cohen's $d = -0.47$. There was no main effect of *Group*, $F(1, 70) = 0.64$, $p = .425$, $\eta^2_p = 0.009$, and no *Group * Stimulus Condition* interaction, $F(1.5, 107.3) = 0.002$, $p = .994$, $\eta^2_p < 0.001$, on mean perceived intensity in attend face trials. The main effect of *Stimulus Condition* on mean perceived intensity in attend face trials was highly significant, $F(1.5, 107.3) = 14.47$, $p <$

.001, η^2_p = 0.17, indicating an incongruency interference effect on intensity ratings in angry face stimuli, *t*(71) = -3.30, *p* = .005, Cohen's *d* = -0.39.

Separate *Group* * *Stimulus Condition* mixed ANOVAs in attend voice trials with target emotion angry revealed no main effect of *Group* on mean RT, *F*(1, 70) = 0.09, *p* = .769, η^2_p = 0.001. There was a highly significant main effect of *Stimulus Condition*, *F*(2, 140) = 20.76, *p* < .001, η^2_p = 0.23, and a significant *Group* * *Stimulus Condition* interaction, *F*(2, 140) = 3.24, *p* = .042, η^2_p = 0.04, on mean RT in attend voice trials. Post-hoc comparisons revealed similar mean RT for both groups in unimodal, *t*(70) = 0.70, *p* = .487, Cohen's *d* = 0.17, congruent, *t*(70) = -0.6, *p* = .549, Cohen's *d* = -0.14, and incongruent angry voice trials, *t*(70) = 0.78, *p* = .438, Cohen's *d* = 0.18. The high proneness group showed an incongruency interference, *t*(35) = 4.21, *p* < .001, Cohen's *d* = 0.7, but no congruency facilitation effect, *t*(35) = -1.02, *p* = .314, Cohen's *d* = -0.17, on mean RT in angry voice trials, whereas the low proneness group showed an incongruency interference effect, *t*(35) = 4.47, *p* < .001, Cohen's *d* = 0.74, and a non-significant trend for higher mean RT in congruent compared to unimodal angry voice trials, *t*(35) = 2.03, *p* = .051, Cohen's *d* = 0.34. A mixed ANOVA on accuracy in attend voice trials could not be interpreted due to violations of homogeneity of error variances (Levene-test *p* < .05). Following a recommendation by Hsu [4], post-hoc comparisons are reported. Post-hoc comparisons revealed lower accuracy in the high proneness (*M* = 78.88, *SD* = 10.72) compared to the low proneness group (*M* = 84.51, *SD* = 6.93) during categorization of angry voice stimuli, *t*(59.89) = -2.64, *p* = .010, Cohen's *d* = -0.62. A congruency facilitation effect on accuracy could be observed in angry voice stimuli, *t*(71) = 17.02, *p* < .001, Cohen's *d* = 2.01. Further, there was no main effect of *Group*, *F*(1, 70) = 0.86, *p* = .356, η^2_p = 0.01, and no *Group* * *Stimulus Condition* interaction, *F*(2, 140) = 0.39, *p* = .68, η^2_p = 0.005, on mean perceived intensity in attend voice trials. The main effect of *Stimulus Condition* on mean perceived intensity in attend voice trials was highly significant, *F*(2, 140) = 39.32, *p* < .001, η^2_p = 0.36, indicating both a congruency facilitation effect, *t*(71) = 3.42, *p* = .003, Cohen's *d* = 0.4, and an incongruency interference effect, *t*(71) = -5.44, *p* < .001, Cohen's *d* = -0.64, on intensity ratings in angry voice stimuli.

In sum, both groups overall showed similar mean RT, accuracy and mean intensity ratings in both attend face and voice trials with target emotion angry, with the exception of accuracy scores in attend voice trials, with the high proneness group being less accurate compared to the low proneness group. Congruency facilitation and/or incongruency interference effects could be

observed for all measures in trials with target emotion angry in both attention conditions, except for mean RT in attend face trials. Overall, both groups showed a comparable pattern of in-/congruency effects for all measures in angry face and voice trials. (see Supplementary Figure S2 second row for mean RT, accuracy and mean perceived intensity for target emotion angry).

Target Emotion Sad. Separate *Group* * *Stimulus Condition* mixed ANOVAs in attend face trials with target emotion sad revealed neither a main effect of *Group*, $F(1, 70) = 1.70$, $p = .197$, $\eta^2_p = 0.02$, nor of *Stimulus Condition*, $F(2, 140) = 1.77$, $p = .175$, $\eta^2_p = 0.03$, and no *Group* * *Stimulus Condition* interaction, $F(2, 140) = 1.12$, $p = .329$, $\eta^2_p = 0.02$, on mean RT. A mixed ANOVA on accuracy in attend face trials was not interpretable due to violations of homogeneity of error variances (Levene-test $p < .05$). Post-hoc comparisons revealed a non-significant trend for lower accuracy in the high proneness ($M = 77.91$, $SD = 10.85$) compared to the low proneness group ($M = 82.25$, $SD = 9.79$) during categorization of sad face stimuli, $t(70) = -1.78$, $p = .079$, Cohen's $d = -0.42$. Both a congruency facilitation, $t(71) = 4.4$, $p < .001$, Cohen's $d = 0.52$, and an incongruency interference effect, $t(71) = -6.5$, $p < .001$, Cohen's $d = -0.77$, could be observed on accuracy in sad face stimuli. Further, there was no main effect of *Group*, $F(1, 70) = 0.01$, $p = .93$, $\eta^2_p = 0.001$, and no Group * *Stimulus Condition* interaction, $F(1.8, 124.4) = 1.61$, $p = .206$, $\eta^2_p = 0.02$, on mean perceived intensity in attend face trials. The main effect of *Stimulus Condition* on mean perceived intensity in attend face trials was highly significant, $F(1.8, 124.4) = 21.03$, $p < .001$, $\eta^2_p = 0.23$, indicating both a congruency facilitation effect, $t(71) = 3.51$, $p = .003$, Cohen's $d = 0.41$, and an incongruency interference effect, $t(71) = -3.6$, $p = .002$, Cohen's $d = -0.43$, on intensity ratings in sad face stimuli.

Separate *Group* * *Stimulus Condition* mixed ANOVAs in attend voice trials with target emotion sad revealed no main effect of *Group* on mean RT, $F(1, 70) = 0.21$, $p = .652$, $\eta^2_p = 0.003$, and no *Group* * *Stimulus Condition* interaction, $F(2, 140) = 0.42$, $p = .959$, $\eta^2_p = 0.001$ (note: there was no homogeneity of covariances in mean RT in sad voice trials, Box-test $p = .001$, not allowing an interpretation of the interaction). There was a highly significant main effect of *Stimulus Condition* on mean RT in attend voice trials, $F(2, 140) = 17.16$, $p < .001$, $\eta^2_p = 0.20$, indicating an incongruency interference effect on mean RT in sad voice stimuli, $t(71) = 5.83$, $p < .001$, Cohen's d= 0.69. A mixed ANOVA on accuracy in attend voice trials was not interpretable due to violations of homogeneity of error variances (Levene-test $p < .05$). Post-hoc comparisons revealed a non-significant trend for lower accuracy in the high proneness ($M = 91.60$, $SD = 7.31$)

compared to the low proneness group (*M* = 94.40, *SD* = 5.66) during categorization of sad voice stimuli, *t*(70) = -1.82, *p* = .073, Cohen's *d* = -.043. An incongruency interference effect on accuracy could be observed in sad voice stimuli, *t*(71) = -6.21, *p* < .001, Cohen's *d* = -0.73. Further, there was no main effect of *Group*, *F*(1, 70) = 0.14, *p* = .705, η^2_p = 0.002, and no *Group* * *Stimulus Condition* interaction, *F*(1.8, 128.3) = 0.15, *p* = .845, η^2_p = 0.002, on mean perceived intensity in attend voice trials. The main effect of *Stimulus Condition* on mean perceived intensity in attend voice trials was highly significant, *F*(1.8, 128.3) = 40.37, *p* < .001, η^2_p = 0.37, indicating an incongruency interference effect on intensity ratings in sad voice stimuli, *t*(71) = -8.71, *p* < .001, Cohen's *d* = -1.03, as well as lower intensity ratings in congruent compared to unimodal sad voice trials, *t*(71) = -3.95, *p* < .001, Cohen's *d* = -0.47.

In sum, both groups showed similar mean RT and mean intensity ratings in trials with target emotion sad in both attention conditions. However, the high proneness group showed a non-significant trend for lower accuracy compared to the low proneness group in trials with target emotion sad in both the attend face and attend voice condition. Congruency facilitation and/or incongruency interference effects could be observed for all measures in trials with target emotion sad in both attention conditions except for mean RT in attend face trials. Both groups showed a similar pattern of in-/congruency effects for all measures in sad face and voice trials (see Supplementary Figure S2 third row for mean RT, accuracy and mean perceived intensity for target emotion sad).

Target Emotion Neutral. Separate *Group* * *Stimulus Condition* mixed ANOVAs in attend face trials with target emotion neutral revealed a non-significant trend for a main effect of *Group* on mean RT, *F*(1, 70) = 3.15, *p* = .081, η^2_p = 0.04, indicating a non-significant trend for lower mean RT in the high proneness (*M* = 1494.04, *SD* = 240.02) compared to the low proneness group (*M* = 1613.13, *SD* = 316.63) when categorizing emotionally neutral face stimuli. There was a highly significant main effect of *Stimulus Condition* on mean RT in attend face trials, *F*(2, 140) = 8.97, *p* < .001, η^2_p = 0.12, indicating a congruency facilitation effect on mean RT in emotionally neutral face stimuli, *t*(71) = -2.49, *p* = .024, Cohen's *d* = -0.29. There was no *Group* * *Stimulus Condition* interaction on mean RT in attend face trials, *F*(2, 140) = 1.79, *p* = .17, η^2_p = 0.03. Further, there was no main effect of *Group*, *F*(1, 70) = 2.59, *p* = .112, η^2_p = 0.04, and no *Group* * *Stimulus Condition* interaction, *F*(1.8, 125.2) = 0.59, *p* = .539, η^2_p = 0.008, on accuracy in attend voice face trials. The main effect of *Stimulus Condition* on accuracy in attend

face trials was highly significant, $F(1.8, 125.2) = 8.64$, $p < .001$, $\eta^2_p = 0.11$, indicating a congruency facilitation effect on accuracy in emotionally neutral face stimuli, $t(71) = 4.06$, $p < .001$, Cohen's $d = 0.48$. There was a significant main effect of *Group* on mean perceived intensity in attend face trials, $F(1, 70) = 6.65$, $p = .012$, $\eta^2_p = 0.09$, indicating significantly higher intensity ratings of emotionally neutral faces in the high proneness ($M = 3.35$, $SD = 0.64$) compared to the low proneness group ($M = 2.91$, $SD = 0.77$). The main effect of *Stimulus Condition* on mean perceived intensity was significant, $F(1.8, 128.8) = 4.48$, $p = .016$, $\eta^2_p = 0.06$, indicating a congruency facilitation effect on intensity ratings in emotionally neutral face stimuli, $t(71) = 3.42$, $p = .003$, Cohen's $d = 0.4$. There was no *Group * Stimulus Condition* interaction on mean perceived intensity in attend face trials, $F(1.8, 128.8) = 0.65$, $p = .512$, $\eta^2_p = 0.009$.

Separate *Group * Stimulus Condition* mixed ANOVAs in attend voice trials with target emotion neutral revealed a non-significant trend for a main effect of *Group* on mean RT, $F(1, 70) = 3.50$, $p = .066$, $\eta^2_p = 0.05$, indicating a non-significant trend for lower mean RT in the high proneness ($M = 1585.00$, $SD = 281.4$) compared to the low proneness group ($M = 1711.25$, $SD = 291.15$) during categorization of emotionally neutral voice stimuli. There was a highly significant main effect of *Stimulus Condition* on mean RT in attend voice trials, $F(2, 140) = 11.84$, $p < .001$, $\eta^2_p = 0.15$. *Group* and *Stimulus Condition* interacted significantly with each other on mean RT, $F(2, 140) = 5.70$, $p = .004$, $\eta^2_p = 0.08$. Post-hoc comparisons revealed lower mean RT in the high proneness compared to the low proneness group in incongruent, $t(70) = -2.98$, $p = .004$, Cohen's $d = -0.7$, but not in unimodal, $t(70) = -0.87$, $p = .414$, Cohen's $d = -0.19$, or congruent neutral voice trials, $t(70) = -1.26$, $p = .212$, Cohen's $d = 0.30$. The high proneness group showed a congruency facilitation, $t(35) = -2.28$, $p = .029$, Cohen's $d = -0.38$, but no incongruency interference effect, $t(35) = -0.41$, $p = .681$, Cohen's $d = -0.07$, on mean RT in neutral voice trials, whereas the low proneness group showed an incongruency interference, $t(35) = 4.2$, $p < .001$, Cohen's $d = 0.7$, but no congruency facilitation effect, $t(35) = -0.9$, $p = .374$, Cohen's $d = -0.15$. There was no main effect of *Group* on accuracy in attend voice trials, $F(1, 70) = 0.07$, $p = .797$, $\eta^2_p = 0.001$. There was a highly significant main effect of *Stimulus Condition*, $F(2, 140) = 11.86$, $p < .001$, $\eta^2_p = 0.15$, and a non-significant trend for a *Group * Stimulus Condition* interaction, $F(2, 140) = 2.83$, $p = .062$, $\eta^2_p = 0.04$, on accuracy in attend voice trials. Post-hoc comparisons revealed similar accuracy for both groups in unimodal, $t(70) = -0.77$, $p = .442$, Cohen's $d = -0.18$, congruent, $t(70) = -1.13$, $p = .262$, Cohen's $d = -0.27$, and incongruent neutral voice trials, $t(70) = 0.97$, $p = .335$, Cohen's $d = 0.23$. The high proneness group showed neither a congruency

facilitation, $t(35) = -0.02$, $p = .982$, Cohen's $d = -.004$, nor an incongruency interference effect, $t(35) = -1.36$, $p = .183$, Cohen's $d = -0.23$, on accuracy in neutral voice trails, whereas the low proneness group showed an incongruency interference, $t(35) = -4.21$, $p < .001$, Cohen's $d = -0.7$, but no congruency facilitation effect, $t(35) = 0.61$, $p = .549$, Cohen's $d = 0.1$. Due to violations of homogeneity of error variances (Levene-test p < .05), a mixed ANOVA on mean perceived intensity was not interpretable. Post-hoc comparisons revealed a non-significant trend for higher intensity ratings of emotionally neutral voice stimuli in the high proneness ($M = 3.32$, $SD = 0.67$) compared to the low-proneness group ($M = 2.98$, $SD = 0.85$), $t(70) = 1.9$, $p = .061$, Cohen's $d =$ 0.45. Further, an incongruency interference effect on mean perceived intensity could be observed in emotionally neutral voice stimuli, $t(71) = -3.14$, $p = .008$, Cohen's $d = -0.37$.

In sum, the high proneness group showed a non-significant trend for lower mean RT in trials with target emotion neutral in both the attend face and attend voice condition as well as higher intensity ratings in attend face and a non-significant trend for higher intensity ratings in attend voice trials with target emotion neutral. Overall, accuracy scores were similar in both groups in trials with target emotion neutral in both attention conditions. Congruency facilitation and/or incongruency interference effects could be observed for all measures in trials with target emotion neutral in both attention conditions. The low proneness group showed a marked incongruency interference effect on mean RT in neutral voice trials, whereas the high proneness group did not show this effect. Further, the high proneness group did not show any in-/congruency effects on accuracy in neutral voice trials, in contrast to the low proneness group (see Supplementary Figure S2 bottom row for mean RT, accuracy and mean perceived intensity for target emotion neutral).

Figure S2

Mean RT, Accuracy and mean Perceived Emotional Intensity for each Target Emotion per Group and Stimulus Condition, Separated by Attention Condition

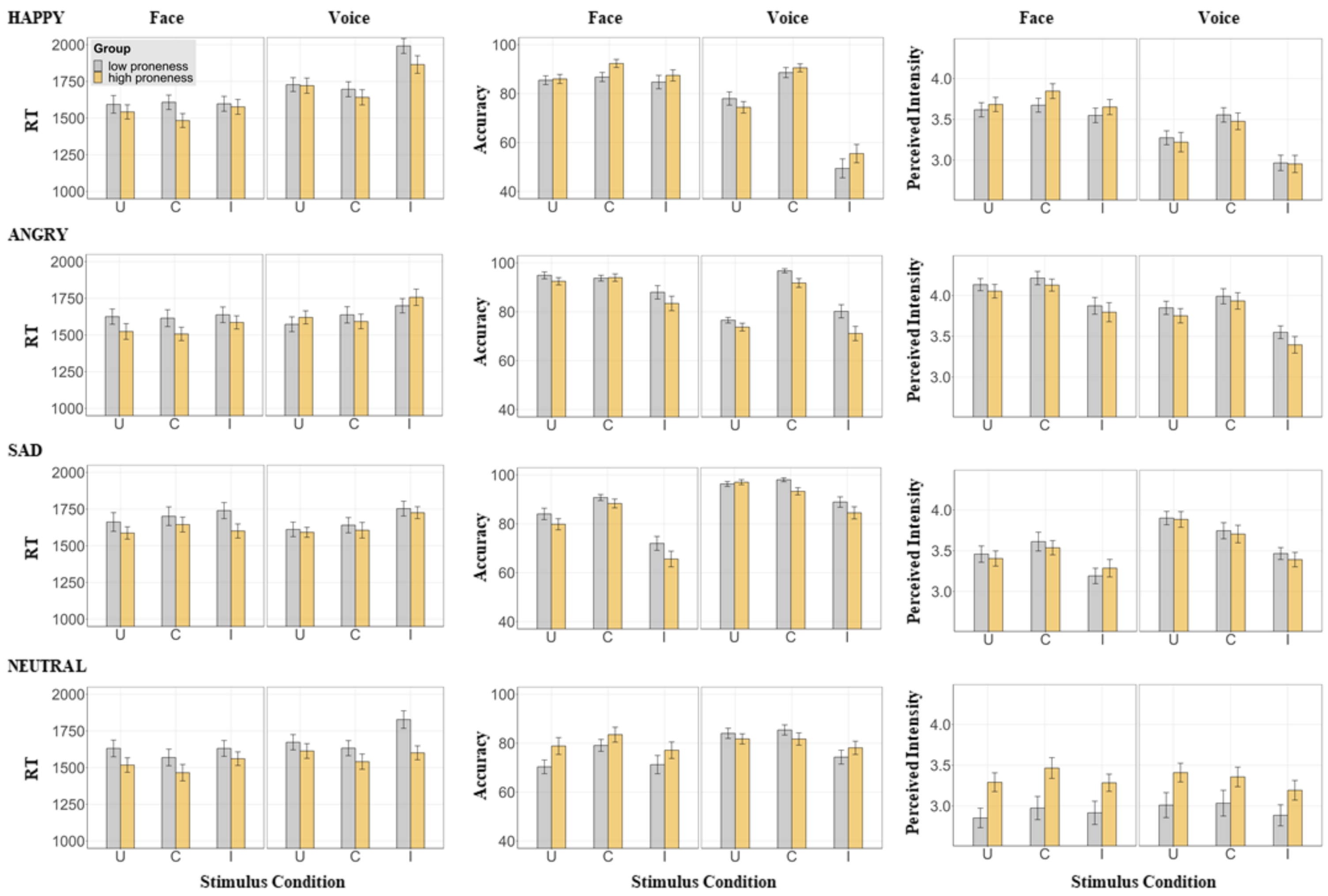

Notes. n_{low} = 36, n_{high} = 36. Error bars denote ±1 standard error of the mean. Face = attend face trials; Voice = attend voice trials; U = unimodal; C = bimodal emotionally congruent; I = bimodal emotionally incongruent.

5 Correlations between Absolute IE Scores and Questionnaire Scores

Pearson correlation coefficients over all subjects indicated negative correlations between IE scores in congruent trials during attend face blocks and both the CAPS (r = -.33, 95% CI [-.52, -.10], uncorrected p = .005) and LSHS (r = -.24, 95% CI [-.45, -.01], uncorrected p = .043), respectively. Neither did survive Bonferroni-correction. No other correlation between IE scores and scores in the CAPS, LSHS or Paranoia Checklist reached statistical significance, all $r < .3$ and $p > .05$ (see Supplementary Table S1 for Pearson's r for correlations between IE scores, CAPS, LSHS, and Paranoia Checklist).

Supplementary Table S1

Pearson Correlation Coefficients r Between Inverse Efficiency Scores and Questionnaire Scores

		1.	2.	3.	4.	5.	6.	7.	8.
Attend Face	1. IE uni								
	2. IE con	**.85*****							
	3. IE inc	**.73*****	**.63*****						
Attend Voice	4. IE uni	**.82*****	**.71*****	**.61*****					
	5. IE con	**.79*****	**.77*****	**.56*****	**.87*****				
	6. IE inc	**.62*****	**.48*****	**.57*****	**.64*****	**.54*****			
	7. CAPS	-.16	**-.33**	-.12	-.05	-.12	-.01		
	8. LSHS-E	-.11	-.24	-.1	-.05	-.14	-.14	**.7*****	
	9. PCL [a]	.09	-.03	.02	.03	.03	.17	**.42*****	**.52*****

Notes. N = 72. Pearson correlation coefficients $r > .3$ are marked in bold. *p*-values are Bonferroni-corrected. IE uni = Inverse Efficiency in unimodal conditions; IE con = Inverse Efficiency in emotionally congruent conditions; IE inc = Inverse Efficiency in emotionally incongruent conditions; CAPS = Cardiff Anomalous Perception Scale, sum of endorsed items; LSHS-E = Launey-Slade Hallucination Scale-Extended, total score; PCL = Paranoia Checklist, total score.

[a] One participant in the low and one in the high proneness group had ≥50% missings in the Paranoia Checklist. Their Paranoia Checklist scores were corrected by the respective group mean.

* $p < .05$, ** $p < .01$, *** $p < .001$

References

1. Addington, J. *et al.* Substance use in clinical high risk for psychosis: a review of the literature: Substance use in clinical high risk for psychosis. *Early Intervention in Psychiatry* **8**, 104–112 (2014).

2. McAusland, L. *et al.* Anxiety in youth at clinical high risk for psychosis: Anxiety in clinical high risk. *Early Intervention in Psychiatry* **11**, 480–487 (2017).

3. Verdoux, H. *et al.* Increased occurrence of depression in psychosis-prone subjects: A follow-up study in primary care settings. *Comprehensive Psychiatry* **40**, 462–468 (1999).

4. Hsu, J. C. (1996). *Multiple comparisons: Theory and methods*. Springer US. https://doi.org/10.1007/978-1-4899-7180-7
